# Production of a reference transcriptome and transcriptomic database (EdwardsiellaBase) for the lined sea anemone, *Edwardsiella lineata*, a parasitic cnidarian

**DOI:** 10.1186/1471-2164-15-71

**Published:** 2014-01-28

**Authors:** Derek J Stefanik, Tristan J Lubinski, Brian R Granger, Allyson L Byrd, Adam M Reitzel, Lukas DeFilippo, Allison Lorenc, John R Finnerty

**Affiliations:** 1 Department of Biology, Boston University, 5 Cummington Mall, Boston, MA 02215, USA; 2 Bioinformatics Program, Boston University, 24 Cummington Mall, Boston, MA 02215, USA; 3 Department of Biology, University of North Carolina at Charlotte, Charlotte, NC 28223, USA; 4 Marine Program, Boston University, 5 Cummington Street, Boston, MA 02215, USA

## Abstract

**Background:**

The lined sea anemone *Edwardsiella lineata* is an informative model system for evolutionary-developmental studies of parasitism. In this species, it is possible to compare alternate developmental pathways leading from a larva to either a free-living polyp or a vermiform parasite that inhabits the mesoglea of a ctenophore host. Additionally, *E. lineata* is confamilial with the model cnidarian *Nematostella vectensis*, providing an opportunity for comparative genomic, molecular and organismal studies.

**Description:**

We generated a reference transcriptome for *E. lineata* via high-throughput sequencing of RNA isolated from five developmental stages (parasite; parasite-to-larva transition; larva; larva-to-adult transition; adult). The transcriptome comprises 90,440 contigs assembled from >15 billion nucleotides of DNA sequence. Using a molecular clock approach, we estimated the divergence between *E. lineata* and *N. vectensis* at 215–364 million years ago. Based on gene ontology and metabolic pathway analyses and gene family surveys (bHLH-PAS, deiodinases, Fox genes, LIM homeodomains, minicollagens, nuclear receptors, Sox genes, and Wnts), the transcriptome of *E. lineata* is comparable in depth and completeness to *N. vectensis*. Analyses of protein motifs and revealed extensive conservation between the proteins of these two edwardsiid anemones, although we show the NF-κB protein of *E. lineata* reflects the ancestral structure, while the NF-κB protein of *N. vectensis* has undergone a split that separates the DNA-binding domain from the inhibitory domain. All contigs have been deposited in a public database (EdwardsiellaBase), where they may be searched according to contig ID, gene ontology, protein family motif (Pfam), enzyme commission number, and BLAST. The alignment of the raw reads to the contigs can also be visualized via JBrowse.

**Conclusions:**

The transcriptomic data and database described here provide a platform for studying the evolutionary developmental genomics of a derived parasitic life cycle. In addition, these data from *E. lineata* will aid in the interpretation of evolutionary novelties in gene sequence or structure that have been reported for the model cnidarian *N. vectensis* (*e.g*., the split NF-κB locus). Finally, we include custom computational tools to facilitate the annotation of a transcriptome based on high-throughput sequencing data obtained from a “non-model system.”

## Background

Parasitism is arguably the dominant trophic strategy on earth, as the number of parasitic species is thought to exceed the number of free-living species, perhaps by 4-to-1 or more [1,2]. Presumably, every cellular organism is subject to parasitism, and parasites affect their hosts in a number of profound ways. For instance, parasites have helped to drive the evolution of sex [3-5] and immune systems [6]. They can markedly change the behavior of their hosts [7], influence host species’ mating strategies and genetic variation [8,9], and contribute to the decline of locally threatened populations [10,11]. However, despite the prevalence of parasitism and its clear ecological and evolutionary importance, parasitic species are relatively poorly characterized. For example, of the 1.5 million species currently named by taxonomists, less than 1% are known to be parasites [12,13].

The evolution of parasitism from an ancestral free-living state can be accompanied by radical alterations to an organism’s ontogeny, bodyplan, and life history (e.g., polyembryony in parasitoid wasps; [14]). Despite this, relatively few studies have explored the developmental evolution of parasitism, mainly because there are practical and theoretical hurdles to such studies. Foremost, it is often difficult to culture parasites in a laboratory setting, as maintaining an obligate parasite requires co-culture of a suitable host. Furthermore, in long-established obligate parasites, the initial steps in their developmental evolution are often obscured by their lengthy evolutionary divergence from free-living outgroups. Finally, parasites are generally not regarded as “model” systems, since parasitic life cycles are often highly derived and therefore not representative of the ancestral free-living condition in major organismal lineages. However, it has been argued that parasites should be of particular interest to evolutionary-developmental biology precisely because their tight associations with host species create “highly integrated reproductive—developmental—ecological systems” that are persistent through space and time [15].

**Figure 1:**
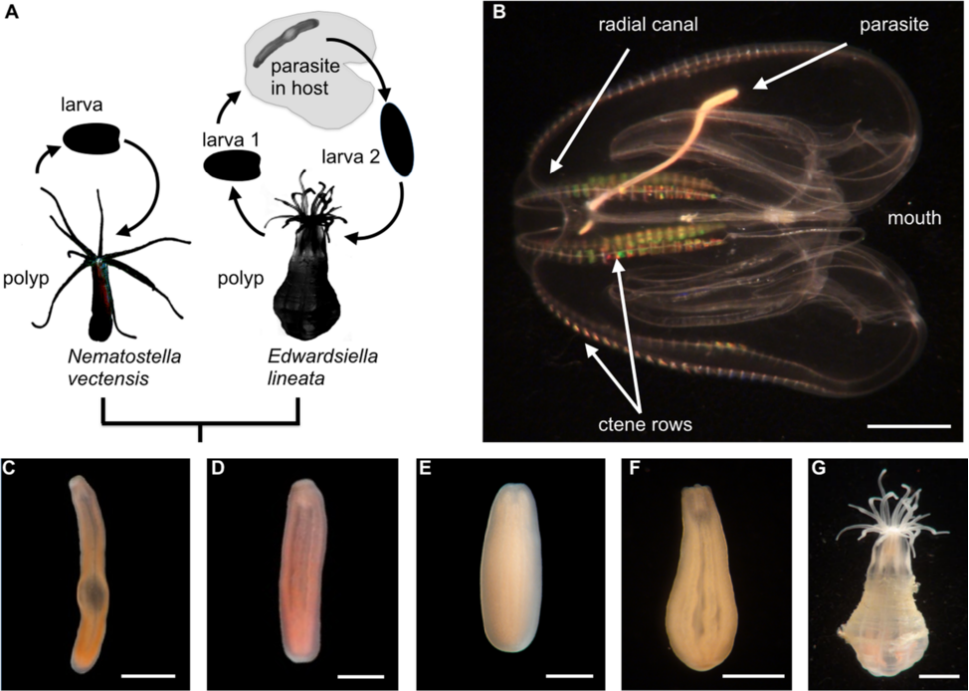
**Life cycle of Edwardsiella lineata. A**. A schematic comparison of the life cycles of the free-living sea anemone *N. vectensis* and the parasitic sea anemone *E. lineata.* Not drawn to scale. **B**. Ctenophore *M.leidyi* infected with parasitic *E. lineata.* Arrow points to parasite’s aboral end. The mouth is located near the junction of the ctenophore’s radial canals. **C-G**. Stages in the life cycle of *E. lineata*. **C**. An excised parasite. **D**. An individual undergoing the transition from the parasite to the post-parasitic larva (larva 2 in panel **A**). **E**. A post-parasitic larva. **F**. An individual undergoing the transition from the post-parasitic larva to polyp. **G**. A polyp. In panels **C-G**., the anemone is oriented with the mouth facing up. *Scale bar:* 5 mm in panel B; 2 mm in panels C,G; 1 mm in panels **D-F**.

The lined sea anemone, *Edwardsiella lineata*, does not exhibit the practical and theoretical limitations that hinder the study of many other parasites, making it a good model for evolutionary developmental studies of parasitism. The larva of *E. lineata* (Figure 1A) parasitizes the pelagic ctenophore *Mnemiopsis leidyi* (Figure 1B; [16]). More than 50% of *M. leidyi* have been found to harbor parasites at Woods Hole, MA, which falls within the native range of the ctenophore [17]. In the North Sea, where the ctenophore has been introduced, up to 6.3% of individuals were found to harbor the parasite [18]. *E. lineata* can enter its host through the body wall or the mouth, eventually coming to reside adjacent to the stomach or one of the eight radial canals that exit the stomach [16,17,19]. When situated in the host, *E. lineata* assumes a novel vermiform body plan (Figure 1C; [20]) and feeds upon the ctenophore’s gut contents. When ready to exit the host, or upon death of the host, the parasite morphs from the elongated form into a planula larva. The planulae are active swimmers, and can follow one of two developmental trajectories, depending on the environment: if presented with a new host, they can re-assume the vermiform body plan of the parasite; however, in the absence of a second host, they can settle and develop into an adult polyp [17]. The polyps live in dense aggregates on the seafloor or on other available hard substrates. Importantly, the parasite is easily collected in infected ctenophores, and it can be maintained indefinitely in the lab as an adult polyp or for several weeks as a parasite inside a ctenophore host (Stefanik, unpublished data). The derived developmental trajectory that leads from the planula to the vermiform parasite can be compared directly to the ancestral anthozoan developmental pathway that leads from the planula to the polyp. Additionally, the ontogeny of *E. lineata* may be compared to that of the starlet sea anemone, *Nematostella vectensis*, which is a leading cnidarian model system for development and genomics [21-24] and a member of the same family as *E. lineata* (Edwardsiidae) [25].

To inform our knowledge of the *E. lineata* gene repertoire, and how changes in expression of particular genes may contribute to ontogenetic changes associated with a derived life history, we sequenced and assembled the transcriptome of *E. lineata* from developmental stage-specific cDNA libraries. We created a database, EdwardsiellaBase, as a platform to share sequence information from *E. lineata* and facilitate queries of gene expression across developmental stages. Both the raw reads and assembled transcriptomic sequences are publicly accessible via the web interface of EdwardsiellaBase.

## Construction and content

### Sequencing and assembly

**Figure 2:**
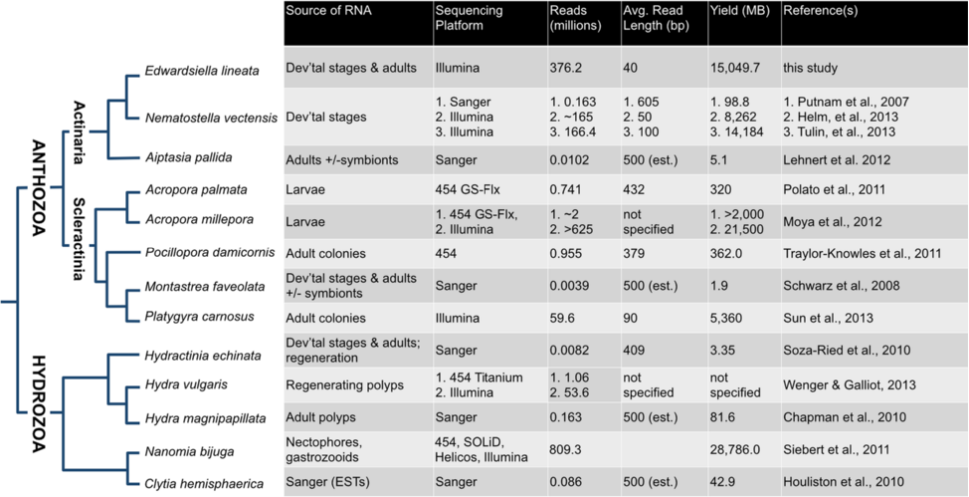
**Published transcriptome sequences for cnidarians.** The methodology and sequencing yield for published cnidarian transcriptomes are summarized here. Taxa are arranged based on their phylogenetic relationships, as compiled from [25,28-32].

Sequencing yielded ~188.1 million read pairs that passed Illumina’s GAIIx quality filter (each read pair consisted of two ~40 nucleotide reads from the same original RNA transcript). The overall sequencing yield of this study (~15,000 MB) exceeded that of all but two published cnidarian transcriptome sequencing projects (Figure 2). The reads were assembled using Velvet [26] and Oases [27] over a range of kmer values (21–39 nucleotides). The assembly comprises 90,440 contigs with an N50 of 1,036 basepairs.

**Figure 3:**
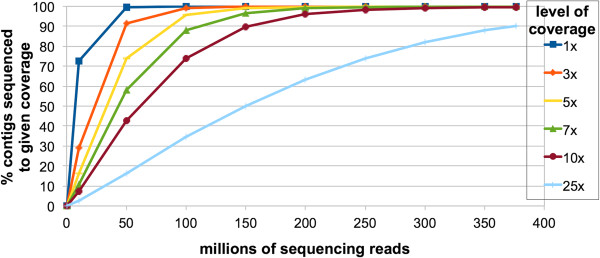
**Sequencing saturation curve.** The percentage of contigs with nominal coverage of n-fold (Y-axis) is plotted against the number of sequencing reads (X-axis). Sequencing sub-samples of a given size were randomly selected from the total pool of sequencing reads. Three replicates were performed for each data point. The mean value is shown. The standard error was too small to represent visually on this graph.

To evaluate whether our sequencing effort provided thorough coverage of the libraries we constructed, we produced a saturation curve that relates the number of sequencing reads to the percentage of the 90,440 contigs covered by those reads (Figure 3). We began to reach saturation at around 250 million sequencing reads. Three replicates of randomly chosen samples of 250 million reads amounted to 10X coverage of 98.4% of the 90,440 contigs.

### Relationship to edwardsiid type specimens

**Figure 4:**
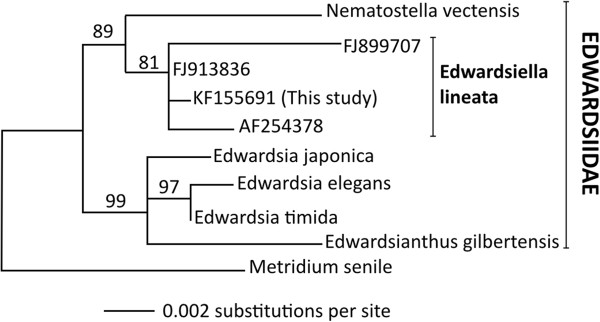
**Phylogeny of edwardsiid 18S sequences.** Maximum likelihood phylogeny of 18S rDNA sequences from 6 edwardsiid anemones and one outgroup taxon, the frilled anemone, *Metridium senile*. Genbank accession numbers are: *Edwardsiella lineata:* (1) this study: KF155691; (2) Daly et al. (2002): AF254378 [33]; (3) voucher SMNH 105142: FJ899707 [18]; (4) voucher SMNH 105141: FJ913836 [18]; *Edwardsia elegans*: AF254376 [33]; *Edwardsia japonica*: GU473304 [34]; *Edwardsia timida*: GU473315 [34]; *Edwardsianthus gilbertensis*: EU190859 [35]; *Metridium senile*: AF052889 [36]; *Nematostella vectensis*: AF254382 [18]. The length of horizontal branches is proportional to the amount of evolutionary change that is inferred to have occurred along that branch; the scale bar at the lower left indicates the number of substitutions per site. Numbers at nodes indicate support for the given clade in 1000 replicates of the bootstrap.

From our transcriptome assembly, we recovered a complete ribosomal RNA transcription unit (18S—ITS1—5.8S—ITS2—28S). We aligned the 18S portion to previously published 18S genes of six species from the family Edwardsiidae and one outgroup taxon (*Metridium senile*; Additional file 1). The edwardsiid data included three previously published 18S sequences from *E. lineata* itself. Maximum likelihood analysis places the sequence obtained in this study in a clade of four *E. lineata* sequences, with bootstrap support of 81% (Figure 4).

### Molecular divergence dating

**Figure 5:**
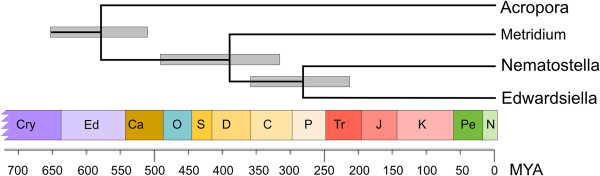
**Estimation of the Nematostella-Edwardsiella divergence.** A portion of a Bayesian phylogenetic tree, based on seven concatenated protein-coding genes and three ribosomal DNAs [37,48], used to date the divergence between *Nematostella* and *Edwardsiella*. The complete analysis comprises 87 taxa (see Methods), but the tree has been pruned so that only the anthozoan clade (corals and sea anemones) is shown. The thick gray bars at each internal node represent the 95% confidence interval for the given divergence time.

We used a molecular clock approach based on seven concatenated protein-coding genes [37] to estimate the divergence date between *E. lineata* and *N. vectensis*. The analysis included seven cnidarians in addition to 81 non-cnidarian taxa for which the full complement of protein sequences is available and robust estimates of divergence times from the fossil record exist ([37-47]; Additional file 2). *N. vectensis* appears as the most closely related taxon to *E. lineata* in the analysis (Figure 5). The divergence time between these two edwardsiid anemones was estimated between 215–364 million years. This compares to an estimated divergence time of 504–652 million years between sea anemones (Actinaria) and hard corals (Scleractinia), which is consistent with a recently published report [28].

### Taxonomic affinity and inferred phylogenetic antiquity of sequences

**Figure 6:**
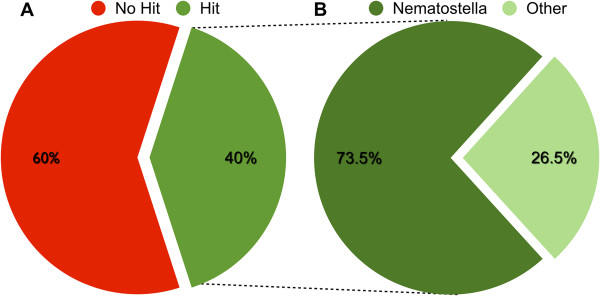
**Summary of BLAST hits. A**. All 90,440 contigs in the assembly were compared to sequences in NCBI’s non-redundant protein database using BLASTx, and 40% produced one or more matches to sequences in the database at a threshold Expect value of −3. **B**. Of the 40% percent of contigs producing BLAST hits, 73.5% had a top hit to a sequence from *N. vectensis.*

Of the 90,440 contigs in our transcriptome assembly, 40% (36,234) produced BLAST hits to sequences in NCBI’s non-redundant (NR) protein database, while 60% (54,206) had no BLAST hits (Figure 6A). Most of the raw reads (>71%) map to those contigs that produce BLAST hits (Additional file 3). Ninety-one percent of the contigs that fail to produce BLAST hits are short (100–500 nucleotides in length; Additional file 4). Nearly three-quarters of the contigs that produced a BLAST hit (73.5%) had a top hit to *N. vectensis* (Figure 6B).

**Figure 7:**
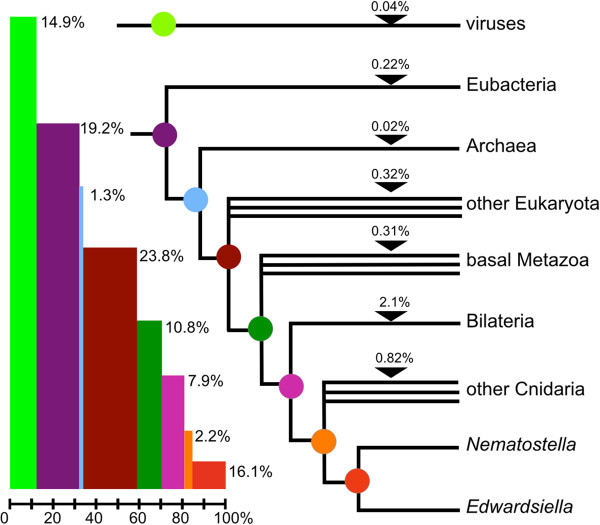
**Inferred phylogenetic antiquity of E. lineata genes.** On the basis of phylogenetically nested BLAST searches, each *E. lineata* contig was tentatively assigned to a particular branch of the phylogeny shown here.

Taxonomically restricted BLAST searches were performed so that we could provisionally ascribe the origin of each of the *E. lineata* transcripts to a particular evolutionary ancestor. For example, a transcript shared with other animal lineages but not non-metazoan eukaryotes or prokaryotes would be assigned to the metazoan ancestor. Using this approach, we infer that 19.2% of the genes producing BLAST hits originated in the common ancestor of Eubacteria and Eukaryotes, another 10.8% originated in the common ancestor of animals, and 2.2% originated in the common ancestor of cnidarians (Figure 7). Sequences producing hits to distantly related lineages, but not to more closely related lineages (e.g., to “Eubacteria” but not to “Bilateria,” “basal Metazoa,” or “other Eukaryota”) probably represent contaminating organisms. Approximately 16% of genes that produced BLAST hits matched only to sequences from the other edwardsiid anemone, *N. vectensis*.

### Gene ontology (GO) analysis

**Figure 8:**
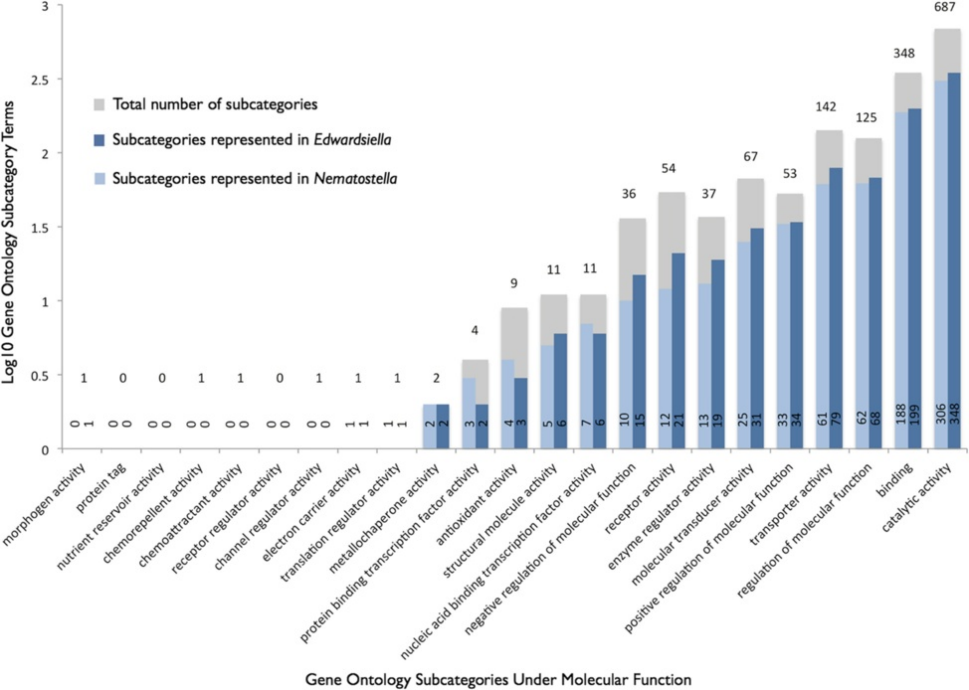
**Recovery of “Molecular Function” gene ontology terms.** Each contig in the *Edwardsiella* transcriptome assembly that produced a BLAST hit was assigned a gene ontology term using Blast2GO. The same analysis was performed for the published EST sequences of *N. vectensis*. The recovery of possible GO terms under each of the primary subcategories of “Molecular Function” is shown here. The bars depict the total number of terms in each subcategory (gray), the number of subcategories recovered in *E. lineata* (dark blue), and the number of subcategories recovered in *N. vectensis* using a Log scale. The absolute numbers are provided on or above each bar.

Of the approximately 40% of contigs (36,234) that produced a BLAST hit to a protein sequence in the non-redundant database at NCBI, roughly half (18,613) could be associated with one or more GO annotation terms. In total, these contigs matched 4,246 GO terms. Because most contigs match many GO terms, there are a total of 244,321 pairings between contigs and GO terms. Using an in-house script (Additional file 5), we tallied the number of matches to the GO terms in the most inclusive subcategories under “Molecular Function” (Figure 8), “Biological Process” and “Cellular Component” (Additional file 6). To place these results in context, we performed the same GO analysis on published ESTs from *N. vectensis*[49]. In general, there was a close correspondence between the recovery of particular GO categories in these two edwardsiid sea anemones. In the 20 categories under “Molecular Function” where a match was possible, we recovered a match from one or both of the anemones for 17 GO categories. For these 17 GO categories, we retrieved an equal number of hits for both anemones in three instances, a slightly greater number of hits for *N. vectensis* in three instances, and a greater number of hits for *E. lineata* in 11 instances. With respect to Molecular Function (Figure 8), the recovery of a greater number of hits in *E. lineata* versus *N. vectensis* was most pronounced for “negative regulation of molecular function” (15 for *E. lineata* vs. 10 for *N. vectensis*), “receptor activity” (21 vs. 12), and “enzyme regulator activity” (19 vs. 13).

### Metabolic pathway analysis

**Figure 9:**
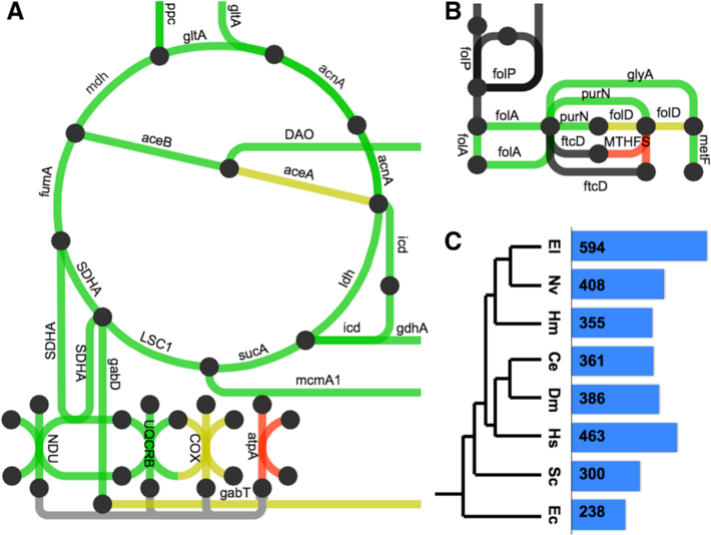
**Recovery of metabolic pathway components.** The networks shown above depict **A**. the Krebs cycle, and **B**. the folate pathway as represented by iPath. The nodes represent metabolites, and the edges represent metabolic transformations. Green edges indicate pathways that were found in both *N. vectensis* and *E. lineata*. Red pathways were only found in *N. vectensis*, and yellow pathways were only found in *E. lineata*. Gray and black edges indicate pathways that were not found in either anemone, in the case of gray edges because no Enzyme Commission numbers map to these edges, and thus they were impossible to detect in our analysis. List of gene name abbreviations for panels A and C are as follow: gltA = citrate synthase; mdh = malate dehydrogenase; aceB = malate synthase A; DAO = D-amino-acid oxidase; aceA = isocitrate lyase; gdhA = glutamate dehydrogenase; sucA = 2-oxoglutarate decarboxylase; LSC1 = succinate-CoA ligase; mcmA1 = methylmalonyl-CoA mutase N-terminal domain; SDHA = succinate dehydrogenase complex, subunit A; gabD = succinate-semialdehyde dehydrogenase I; UQCRB = ubiquinol-cytochrome c reductase binding protein; folP = dihydropteroate synthase; glyA = serine hydroxymethyltransferase; purN = phosphoribosylglycinamide formyltransferase; metF = 5,10-methylenetetrahydrofolate reductase; MTHFS = 5,10-methenyltetrahydrofolate synthetase; ftcD = glutamate formiminotransferase; folA = dihydrofolate reductase; folD = bifunctional 5,10-methylene-tetrahydrofolate dehydrogenase; ppc = phosphoenolpyruvate carboxylase; acnA = aconitate hydratase 1; icd - isocitrate dehydrogenase; ldh = L-lactate dehydrogenase; fumA = fumarate hydratase; COX = Cytochrome c oxidase; atpA = ATP synthase subunit alpha; gabT = 4-aminobutyrate aminotransferase; NDU = NADH dehydrogenase. **C**. A selection of species that share ancestry with *E. lineata* at various evolutionary distance. The bar graph and numbers represent the amount of shared EC numbers between that species and *Edwardsiella lineata*. The species are *E. coli*, *S. cereviseae*, *H. sapiens*, *D. melanogaster*, *C. elegans*, *H. magnipapillata*, *N. vectensis*, and *E. lineata*.

To identify metabolic pathways represented by the assembled contigs, we extracted the Enzyme Commission (EC) numbers from our Blast2GO results for *E. lineata*. We then cross-referenced these with EC numbers already assigned by The Kyoto Encyclopedia of Genes and Genomes (KEGG; [50]) to predicted genes in *N. vectensis*. Overall, there are 5935 EC numbers, of which, 638 are associated with *N. vectensis*. One or more EC numbers could be associated with 2,148 of the *E. lineata* contigs. These contigs produced matches to 594 EC numbers, of which, 408 are shared between *N. vectensis* and *E. lineata*, while 186 were found in *E. lineata* but not *N. vectensis*. The metabolic pathways represented by the *E. lineata* contigs and *N. vectensis* predicted genes were diagrammed using iPath 2.0 (Figure 9; Additional file 7; [51]).

### Recovery of specific genes and gene families from *E. lineata*

To evaluate the comprehensiveness of this transcriptome, we searched for *E. lineata* representatives of eight different gene families that have already been surveyed in *N. vectensis*[52-59]: bHLH-PAS, deiodinases, Fox genes, LIM homeodomains, minicollagens, nuclear receptors, Sox genes, and Wnts. We also sought to identify the transcription factor NF-κB among the *E. lineata* contigs, because a number of functional studies have been performed on NF-κB in *N. vectensis*[60-63], and the overall structure of the protein in this species [64] appears to be derived relative to the ancestral condition for metazoans [65]. To identify members of these gene families in *E. lineata*, we used the known *N. vectensis* sequences to query the *E. lineata* transcriptome using reciprocal BLAST searches (see Methods). Our searches recovered an equivalent or nearly equivalent number of gene family members in *E. lineata* as had been previously reported for *N. vectensis* (Table 1).

**Table 1:** Recovery of gene family members from Edwardsiella

		***N. vectensis***		
**Gene family**	***E. lineata***	**Published studies**^**1**^	**ESTs**^**2**^	**Human**
bHLH-PAS	7	7 [48]	7	11
Deiodinase	5	4 [49]	1	3
Fox	17	14 [50]	16	42
LIM homeodomain	6	6 [44,45]	4	12
Minicollagens	3	5 [47]	5	-
Nuclear receptors	10	17 [46]	12	48
Sox	12	14 [50]	9	20
WNT	13	12 [43]	7	19

^1^Published studies on individual gene families; ^2^Expressed sequence tags generated as part of the Nematostella genome sequencing project [49].

**Figure 10:**
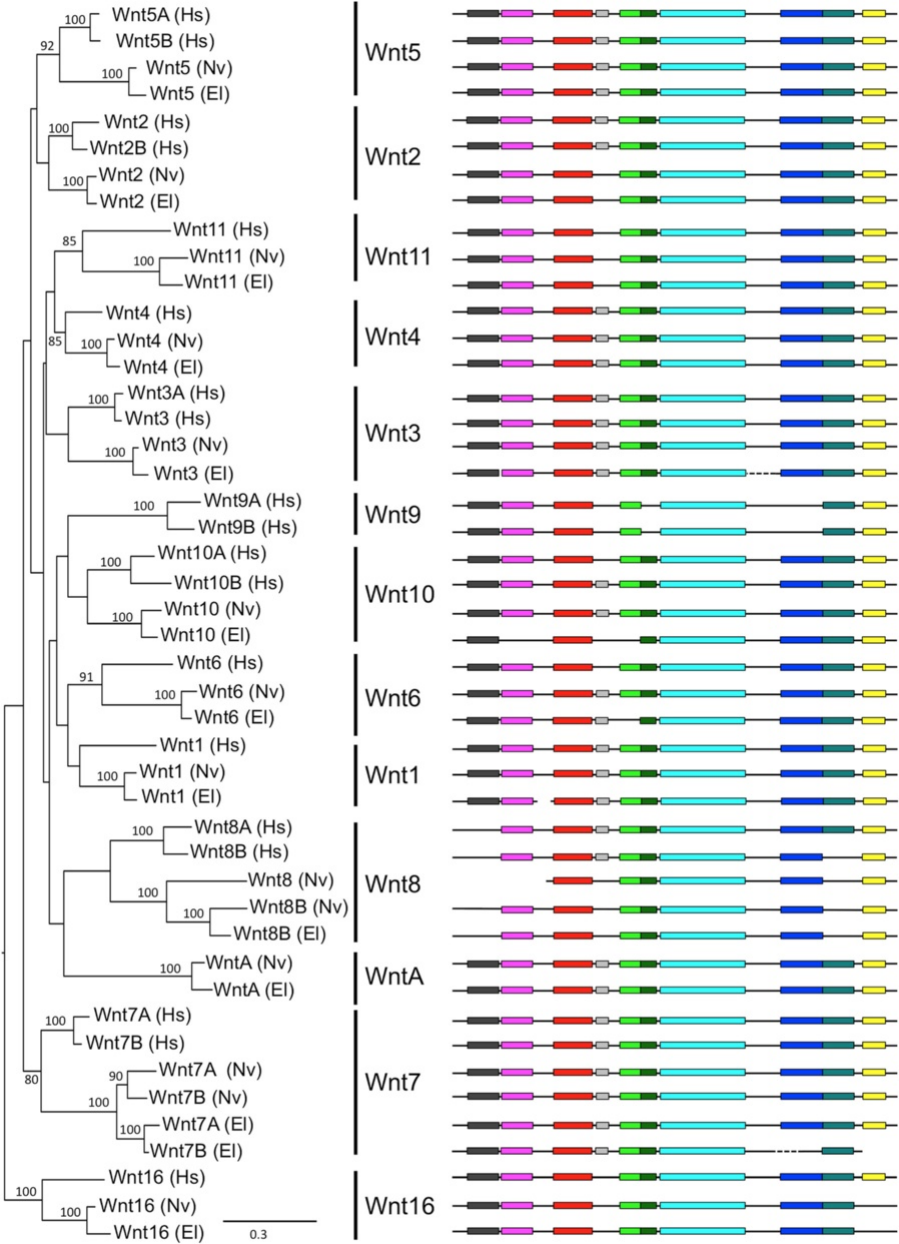
**Maximum likelihood tree of Wnt genes.** The tree shown is based on a maximum likelihood analysis of an amino acid alignment of the Wnt consensus motif (PF00110). Numbers at nodes represent bootstrap values above 80%. Branch length is shown in terms of expected number of substitutions per residue (bar at lower right). Conserved motifs were identified using MEME, as described in the methods. Motifs (colored boxes) are drawn to scale, but the inter-motif regions (black lines) were altered to allow the motifs to align for ease of visualizing conservation in motif composition and order.

To evaluate the phylogenetic relationships among gene family members, we performed maximum likelihood analyses for bHLH-PAS, deiodinases, LIM homeodomains, minicollagens, nuclear receptors and Wnts. With the exception of minicollagens, each gene family analysis was based on protein sequences from deuterostome (human) and cnidarian (*N. vectensis; E. lineata*) lineages. Minicollagens are specific to cnidarians, and therefore the minicollagen tree contains no deuterostome sequences. A phylogeny of Wnt genes is presented in Figure 10 (all other phylogenetic trees are contained in Additional file 8). Based on the phylogenetic analyses, in almost all cases, for each previously reported *N. vectensis* gene, we recovered an *E. lineata* ortholog. For example, in the Wnt phylogeny, both anemones possess representatives of 12 out of 13 Wnt subfamilies, and within each of these subfamilies, the sister-group to a sequence from *N. vectensis* is a sequence from *E. lineata*. The only Wnt subfamily not represented in *E. lineata* or *N. vectensis* is Wnt9. The protein motif analysis (Figure 10) revealed extensive conservation among Wnt proteins from humans and edwardsiid anemones. Of note, all but three of the *E. lineata* Wnt transcripts (the exceptions being Wnt10, Wnt6, and Wnt7B) encode predicted proteins that share all motifs found in their *N. vectensis* orthologs.

**Figure 11:**
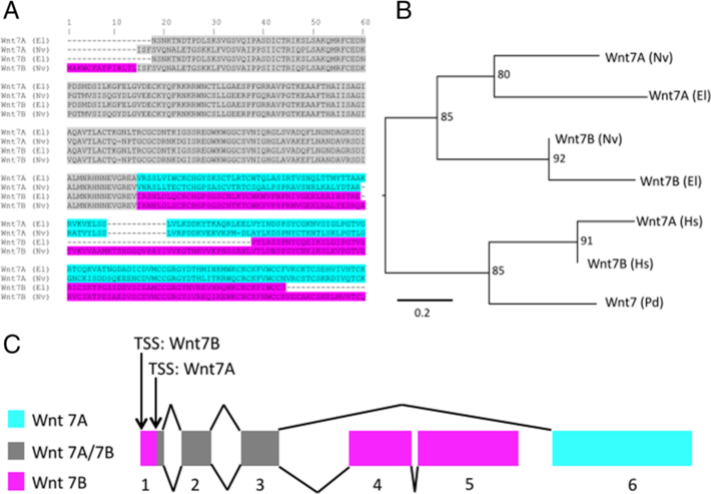
**Wnt7 splice variants in Edwardsiella and Nematostella. A**. Amino acid alignment of Wnt7A and 7B transcripts from *E. lineata* and *N. vectensis*. In the gray region, the amino acid sequence and the underlying nucleotide sequence of *E. lineata* Wnt7A is identical to that of *E. lineata* Wnt7B. Similarly, the amino acid sequence and underlying nucleotide of *N. vectensis* Wnt7A is identical to that of *N. vectensis* Wnt7B. In the regions of the alignment highlighted in blue and pink, the amino acid sequence of *E. lineata* Wnt7A is most similar to *N. vectensis* Wnt7A (blue) and the amino acid sequence of *E. lineata* Wnt7B is most similar to *N. vectensis* Wnt7B. **B**. A maximum likelihood phylogeny based on amino acid sequences of Wnt7A and 7B but excluding the portion of the alignment shared by *E. lineata* Wnt7A and Wnt7B (the region shaded in gray). Numbers at nodes indicate how many times the given clade was recovered in 1000 replications of the bootstrap. The scale bar represents the number of substitutions per site. Taxon abbreviations are as follows: El = *Edwardsiella lineata*; Hs *= Homo sapiens*; Nv = *Nematostella vectensis.***C**. Diagram of the *Nematostella* Wnt7 locus illustrating the similarities and differences of the Wnt7A/7B splice variants (adapted from [66]). Wnt7A is composed of sequences from exons 1b, 2, 3, and 6, and Wnt7B is composed of exons 1, 1b, 2, 3, 4, and 5.

As previously reported for *N. vectensis*[66], we have identified two Wnt7 splice variants in *E. lineata*. In the Wnt phylogeny (Figure 10), the two *N. vectensis* variants (7A and 7A) appear most closely related to each other, as do the two *E. lineata* variants (7A and 7B). This is due to the fact that, within each species, the splice variants share a substantial amount of sequence identity (Figure 11). However, the *N. vectensis* Wnt7A appears to share the same exon composition with *E. lineata* Wnt7A, while the *N. vectensis* Wnt7B shares the same exon structure with *E. lineata* Wnt7B. A phylogenetic analysis of all four sequences based upon only the regions of the protein they share in common groups *N. vectensis* Wnt7A with *E. lineata* Wnt7A and *N. vectensis* Wnt7B with *E. lineata* Wnt7B (Figure 11).

**Figure 12:**
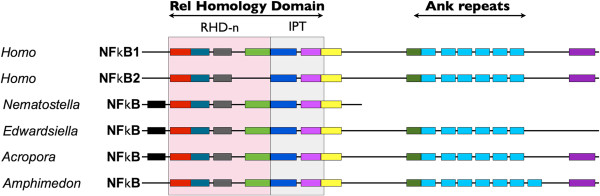
**Conservation and loss of motifs in NF-κB proteins.** Conserved protein motifs were identified using MEME. Motifs (colored boxes) are drawn to scale, but the inter-motif regions (black lines) were altered to allow the motifs to align for ease of visualizing conservation in motif composition and order. The sequences included in the analysis were the NF-κB proteins of three cnidarians (*Acropora millepora*, *E. lineata*, *N. vectensis*) and one sponge (*Amphimedon queenslandica*) as well as the NF-κB1 and NF-κB2 proteins of *Homo sapiens*.

As in *N. vectensis*[64], there appears to be only one NF-κB family member in *E. lineata*. However, unlike *N. vectensis*, the single *E. lineata* NF-κB reflects the ancestral structure in that it contains both an N-terminal Rel Homology Domain (RHD) and a C-terminal inhibitory IκB domain consisting of multiple ankyrin repeats (Figure 12). In *N. vectensis*, the ancestral NF-κB locus is split, so that the RHD and IκB domains are encoded by separate loci [64,65].

## Utility

**Figure 13:**
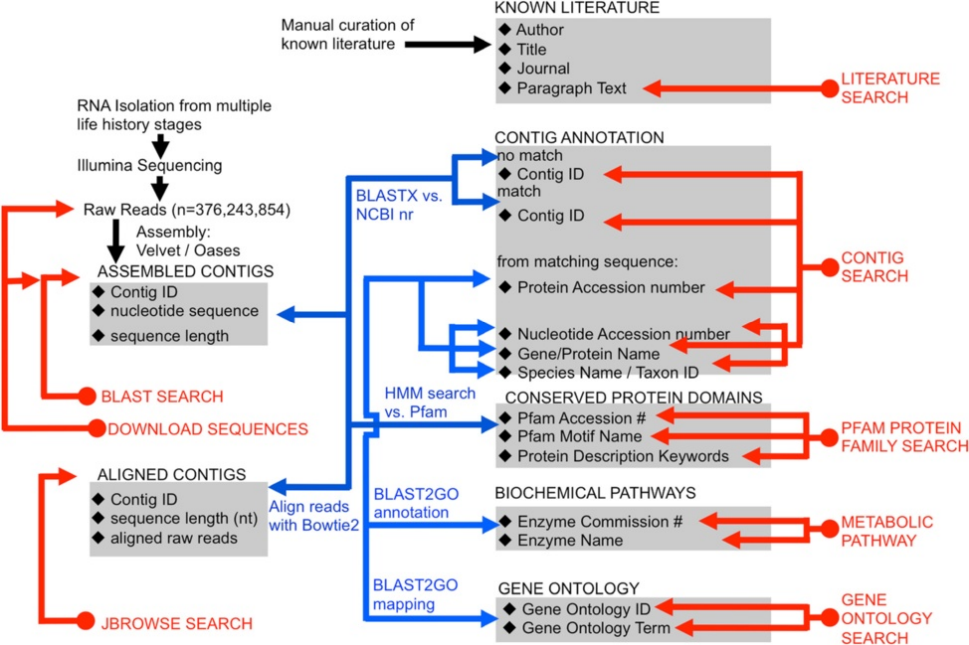
**EdwardsiellaBase data sources and queries.***EdwardsiellaBase* houses the assembled contigs that were generated in this study as well as the output from a number of bioinformatic analyses performed on them. The black diamonds indicate all of the searchable fields contained in the database’s tables (gray shading). Blue arrows indicate how the tables were populated, while red arrows indicate how the data may be queried.

The raw sequencing reads and the contigs generated from our transcriptome assembly are housed at EdwardsiellaBase (http://www.EdwardsiellaBase.org), whose overall organization is based on PocilloporaBase [67]. The database was populated as follows (Figure 13; blue arrows). Each of the assembled contigs is associated with a Contig ID, nucleotide sequence, and sequence length. Those contigs that produced a BLAST hit at NCBI are also associated with the protein accession numbers from the top five hits, and these numbers were used to retrieve additional information from NCBI (Gene/Protein Name and Species Name/Taxon ID). Then, using Blast2GO, the protein accession numbers were used to retrieve information about biochemical pathways (Enzyme Commission Number; Enzyme Name) and gene ontology (Gene Ontology ID; Gene Ontology Term). All contigs were translated in all six frames and searched using HMMer to identify conserved protein domains (Pfam Accession Number; Pfam Motif Name; Pfam Description Keyword). The raw reads were aligned to the assembled contigs using Bowtie 2 (v. 2.0.0-beta; [68]).

The data can be searched by Contig, Protein Family, Metabolic Pathway or Gene Ontology (Figure 13; red arrows). EdwardsiellaBase also supports the complete range of BLAST options to search the assembled contigs for matches to a query sequence. Finally, the JBrowse [69,70] function enables one to view alignments of the raw reads to the assembled transcriptome to help assess validity of transcripts. A literature database allows users to search the published literature on *Edwardsiella* using matches to keywords or any user-entered text string. The database structure and entity relationships are depicted in Additional file 9.

## Discussion

### Evidence that the transcriptome is representative

The present study describes a transcriptome assembly for *E. lineata* based on roughly 15 billion nucleotides of RNA sequencing. This is one of the largest transcriptomic datasets currently available for any cnidarian [28,67,71-85], and approximately 2.5 times the sequencing yield estimated to be sufficient for assembling a representative transcriptome [86]. To ensure that we captured transcripts expressed throughout *E. lineata’s* complex life cycle, we generated cDNA libraries from five distinct developmental stages. Our saturation analysis showed that (Figure 3) additional sequencing of these libraries would result in identification of relatively few novel transcripts. Evidence that the transcriptome assembly is representative of the expressed gene repertoire of an edwardsiid anemone is the comparable recovery of GO terms (Figure 8; Additional file 6) and gene families (Table 1) (Figures 10, 11 and 12; Additional file 6) from *E. lineata* and *N. vectensis*. Taken together, these data suggests that our sequencing effort was sufficient to produce a representative transcriptome that captures a large fraction of the transcript variety encoded by the *E. lineata* genome. Undoubtedly, we have failed to capture some transcripts that are expressed at very low levels during the developmental stages studied here, or that are expressed only in different developmental, physiological, or environmental contexts.

### Utility of *E. lineata* for comparative transcriptomics and genomics

The utility of any species for comparative transcriptomic and genomic studies depends on its relationship to other taxa for which extensive sequence data are available. Molecular, morphological, and developmental characters support the placement of *E. lineata* within the family Edwardsiidae and the subfamily Milneedwardsiinae, a clade comprising the genera *Edwardsiella, Nematostella, Drillactis*, and *Paraedwardsia*[25,33,87]. The 18S phylogenetic analysis performed here confirms the specimens we characterized as *E. lineata.* This confirmation is important, given that we are seeking to establish a reference transcriptome for the species. The 18S phylogenetic analysis also supports the placement of *Edwardsiella* and *Nematostella* within the Milneedwardsiinae (Figure 4). Thus, this study supports the conclusion that *E. lineata* is one of the closest living relatives of *N. vectensis*. Our molecular clock estimate (Figure 5) suggests the divergence between *Nematostella* and *Edwardsiella* occurred sometime between the early Triassic Period (215 mya) and the early Devonian (>360 mya). As *N. vectensis* protein-coding genes appear to evolve at a rate comparable to, or even slower than vertebrates [49], the evolutionary distance between *Edwardsiella* and *Nematostella* is likely sufficient to facilitate the identification of functional conservation in protein sequence and structure; *i.e*., at this distance, sequence conservation is not likely to reflect mere phylogenetic inertia. Looking forward, comparing genome sequences between these two edwardsiid anemones is likely to be useful in identifying conserved cis-regulatory sequences, as has been done for echinoderm species spanning divergences from 35–500 mya [88,89].

### BLAST based annotation

Forty percent of the assembled contigs in the *E. lineata* transcriptome produced BLAST hits to sequences in NCBI’s non-redundant (NR) protein database, while 60% did not match any protein sequences in the database (Figure 6). This ratio between BLAST hits and misses for contigs within the *E. lineata* transcriptome is comparable to another published cnidarian transcriptome assembly for the coral *Pocillopora damicornis*[67]. The high percentage of contigs in the *E. lineata* assembly that do not produce BLAST hits may be a function of contig size. Ninety-one percent of the contigs that fail to produce BLAST hits are relatively short (100–500 nucleotides in length; Additional file 4). Since BLAST scores are influenced by sequence match length, shorter sequences will produce lower scores, and may also be more likely to represent assembly artifacts or truncated transcript models. Over two-thirds of the raw reads (>71%) map to contigs that produce BLAST hits (Additional file 3).

Another explanation for the presence of contigs in the *E. lineata* transcriptome assembly that produced no BLAST hits to NR protein database is that some of the contigs may represent assembly of long, non-coding RNA transcripts, for which no cognate protein would exist in the NR database. We used BLASTn to query the NONCODE database [90] with the set of contigs that produced no hits against the NR protein database. This search yielded matches for 354 contigs. The *E. lineata* transcriptome assembly therefore contains non-coding transcripts, but these transcripts represent a small fraction of the total contigs that produced no BLAST hits to the NR protein database.

Given the key position of cnidarians in metazoan phylogeny — as the likely sister group to triploblastic bilaterians — there is widespread interest in pinpointing the evolutionary origin of cnidarian genes. For example, which genes have been conserved since the time of the eumetazoan common ancestor, and which genes are cnidarian inventions? We approached this question using taxonomically restricted BLAST searches (Figure 7). Using this approach, we can ascribe putative origins to the genes that encode the *E. lineata* transcripts we recovered. For example, 19.2% of the *E. lineata* contigs generated significant matches to sequences from other Eukaryota, plus Eubacteria, and Archaea, suggesting (1) that these genes originated prior to the origin of Eukaryota, and (2) they have been conserved in eukaryotes and prokaryotes since that time. The number of hits produced from this analysis can be influenced by a few confounding factors, which should be considered when viewing the results. While it is possible that these sequences represent shared transcripts of essential function common to the organisms to which we ascribed their origin and their descendent lineages; a potentially confounding variable is that it is also possible that some of these sequences are transcripts produced by other organisms residing within and/or on the focal taxon, and which were subsequently sequenced and deposited in the nr database, or represent unintended taxonomic sampling from the holobiont of the anemone in this study. Due to potentially confounding factors, and the relatively permissive BLAST cutoff threshold utilized, the analysis of taxonomic affinity in this study represents a provisional phylogenetic stratigraphy of gene origins. To achieve a more robust assignment of origin across the taxonomic breadth of this study, one would need to produce multiple sequence alignment and phylogenetic trees for each of the 90,440 transcripts in the *E. lineata* transcriptome.

The BLAST-based approach used here is currently limited by the uneven representation of major taxonomic groups in the NCBI database, including the phylum Cnidaria. While over 16% of the *E. lineata* sequences generated significant matches to *N. vectensis* alone, only 2.2% generated matches to other cnidarians in addition to *Nematostella*. This disparity is likely a reflection of the relatively large amount of data from *N. vectensis* in the database. As more cnidarian taxa are sequenced, we expect many of the sequences from *E. lineata* that currently generate hits to *Nematostella* alone will be shared across the phylum.

### Gene ontology (GO) analysis

We were able to assign 17 GO subcategory terms under the “Molecular Function” ontology to transcripts from either the *N. vectensis* ESTs and *E. lineata* sequencing produced from this study (Figure 8). Sixteen of these subcategories were represented in transcripts from both sea anemones. However, the Molecular Function subcategory of “morphogen activity” was only assigned to sequences from *E. lineata.* Of the remaining 16 subcategories, there is a generally close correspondence in presence/absence of subcategories within each ontology between the expressed sequence resources from each sea anemone. Taken together, these findings suggest that the transcriptome assembly produced for *E. lineata* is comparably representative of the expressed transcript repertoire of an edwardsiid sea anemone as the *N. vectensis* ESTs. This interpretation is based on the assumption that these two confamilial sea anemones would exhibit similar gene ontology distributions in their expressed transcripts as a function of shared, derived physiological and genomic characteristics.

### Recovery of selected gene families in *E. lineata*

The largely consistent recovery of orthologous genes from seven divergent gene families in *E. lineata* and *N. vectensis* suggests that the genetic repertoire of these two edwardsiid anemones is well conserved and that the reference assembly described here provides thorough coverage of the *E. lineata* transcriptome. Figure 10 depicts a Maximum Likelihood phylogenetic analysis of Wnt sequences from *E. lineata, N. vectensis,* and human, alongside a MEME analysis of the protein coding domains of these transcripts. This analysis reveals extensively conserved protein motif architecture across Wnt proteins between cnidarians and human (the deuterostome representative). Additionally, motif conservation is high between the two sea anemones, with the entire motif complement for each protein being conserved between *N. vectensis* and *E. lineata*, with the exception of three transcripts (Wnt10, Wnt6, and Wnt7B) in which one or more motifs are discordant between the two taxa. All *E. lineata* sequences used in this analysis represent single contigs (with the exception of Wnt3, Wnt1 and Wnt7B, which were conceptually spliced). Taken together with the degree of protein coding motif conservation between the two sea anemones, this suggests that many contigs represent full-length transcripts. The detailed analysis of Wnt7 sequences (Figure 11) also clearly supports the conclusion that the Wnt7A/7B splice variants are conserved between *N. vectensis* and *E. lineata*.

### No evidence for pervasive change in the gene repertoire of this parasite

This study has produced no evidence for pervasive changes in the gene repertoire of *E. lineata* that might have evolved in concert with the evolution of its novel parasitic life cycle. In contrast, a recent study on four cestodes identified extensive losses of genes and pathways that are broadly conserved in other animals as well as the origin of specialized metabolic pathways adapted to extract nutrients from the host [91]. This is to be expected given that cestodes are an ancient lineage of obligate internal parasites. Although we cannot date the antiquity of parasitism in *E. lineata*, except to say that it must postdate the last common ancestor with *N. vectensis,* we should not expect extensive gene losses in *E. lineata*, as this parasitic anemone retains all of the life cycle stages present in related free-living anemones. Therefore, it would presumably require the same developmental regulatory genes and metabolic pathways. Despite its derived life cycle, we expect that there will be genes and proteins for which *E. lineata* reflects the primitive condition, while the free-living *N. vectensis,* an important cnidarian model system, exhibits a derived condition. NF-κB is such an example, as the NF-κB protein of *E. lineata* reflects the ancestral protein structure, in which the DNA-binding domain and inhibitory domain are contained within the same transcript, whereas these domains are split between two separate loci in *N. vectensis* (Figure 12)*.* As an interesting aside, NF-κB appears to be one of the genes lost in parasitic cestodes [91]. We expect that *E. lineata* has evolved some genetic modifications that would make it better able to exploit its host ctenophore, though these may be few in number. A detailed analysis of differential gene expression between developmental stages, which is beyond the scope of this paper, is currently underway.

### Functionality of *EdwardsiellaBase*

EdwardsiellaBase was modeled after the previously published species-specific cnidarian databases PocilloporaBase [67], and StellaBase [92,93], but it expands upon their functionality in key ways. As with these published databases, an html-based interface allows users to search the assembled contigs using contig identifiers, enzyme names or EC numbers, protein families (Pfam), protein names, and Gene Ontology (GO) information (Figure 13). The database also features a fully equipped BLAST interface for searching the assembled contigs based on sequence similarity to known genes and proteins. New functions include a literature search, JBrowse alignment viewer [69,70], and individual contig pages. The literature search allows the user to query the *E. lineata* literature, much of which has been published in relatively inaccessible venues, such as books that are out of print. The JBrowse feature allows users to view alignments of reads to assembled contigs to and visualize the relative abundance of transcripts, including alternate splice forms. The individual contig page summarizes available information, and also provides a notes section, to which users can submit entries. Provisional gene names have been assigned to each contig that produced a BLAST hit using Blast2GO. The database may be searched using these gene names, and when a name has been assigned to a given contig, that name is provided on the contig information page.

It is also possible to search for matches to a query sequence using the complete set of BLAST options. BLAST searches return a standard BLAST page, with a few additional features.

## Conclusions

We describe the sequencing and assembly of a reference transcriptome for the parasitic cnidarian, the lined sea anemone, *E. lineata*. This dataset represents a significant contribution to the comparative study of cnidarian transcriptomes because of (1) the overall sequencing yield (~15,000 Mb of nucleotide sequence), (2) the phylogenetic placement of *E. lineata* as the closest cnidarian taxa to *N. vectensis* for which appreciable molecular sequence data exist, and (3) the fact that *E. lineata* is a recently evolved parasite whose novel life cycle is tractable to laboratory investigation. The assembled transcripts published in this study capture the large majority of the transcriptome of this sea anemone. The diversity of Gene Ontology terms, metabolic pathways components, and gene family members we were able to recover from the *E. lineata* contigs compares favorably with published EST data from *N. vectensis*. The assembled contigs are available in a searchable database, EdwardsiellaBase, that will serve as a platform for studying the evolutionary developmental genomics of *E. lineata’s* novel, derived parasitic life history, and will be useful for comparative transcriptomic studies between cnidarian taxa, particularly between *E. lineata* and *N. vectensis*. The scripts and computational tools employed in this study are included in the supplementary files to facilitate the annotation of transcriptome assemblies from other emerging model systems for which genomic data are not available.

## Availability and requirements

EdwardsiellaBase is freely available at http://edwardsiellabase.org.

## Methods

### Animal collection and developmental sampling

Ctenophores (*Mnemiopsis leidyi*) infected with *E. lineata* were collected from July through October of 2009 and 2010 at Woods Hole, MA as previously described [17]. Approximately two-hundred *E. lineata* parasites were extracted from approximately 70 *M. leidyi* using forceps and a scalpel. Approximately 30 of these excised parasites (Figure 1C) were immediately harvested for RNA isolation. The remaining parasites were transferred to full-strength artificial seawater (Instant Ocean; salinity = 36 parts per thousand) and maintained at room temperature, so they could continue their development [17]. Individuals were then selected to represent particular developmental stages based on the duration of their incubation and their gross morphological appearance. To represent the parasite-to-planula transition stage (Figure 1D), approximately 30 of the developing anemones were collected for RNA isolation 12–24 h after their excision from the host. The anemones at this stage of development exhibited the following three phenotypic and/or behavioral criteria: (1) reduction in pharynx length relative to the parasitic stage, (2) ability to move via cilia, and (3) an overall body shape that was intermediate between the vermiform parasite and the ovoid planula. To represent the larval stage (the planula; Figure 1E), approximately 30 anemones were allowed to develop for 2–4 days post host excision. The planulae exhibited the following characteristics: (1) lack of transparency, (2) vigorous swimming ability, and (3) ovoid shape. Thirty of the remaining larvae were allowed to develop until they began showing signs of metamorphosis into polyps (Figure 1F), such as (1) cessation of swimming and (2) tentacle eruption. The adult stage (Figure 1G) was represented by individuals that successfully metamorphosed into polyps capable of using their tentacles to feed on freshly hatched brine shrimp larvae (*Artemia salina*). Six individuals were harvested for RNA isolation at this stage.

### RNA isolation, library preparation and sequencing

Total RNA was isolated from pooled specimens for each of the five developmental stages (Figure 1C-G). For the four pre-adult stages (parasites, the parasite-to-larva transition, larvae, and the larva-to-polyp transition), we used ~30 individuals in each case, which is equivalent to ~100 mg of tissue. For the adult polyp we isolated RNA from 6 individuals. For the pre-adult stages, total RNA was isolated using TRIzol (Life Technologies) according to the manufacturers protocol. From adult polyps, total RNA was isolated using the Omega Biotek Mollusk RNA Isolation Kit. Subsequently, mRNA was isolated from each pool of total RNA using the Poly(A) Purist mRNA isolation kit (Ambion). Separate cDNA libraries were prepared for each of the five developmental stages using the mRNA Sample Preparation Kit from Illumina. Sequencing of cDNA libraries was performed on a Genome Analyzer IIx (Illumina). Each library was sequenced on an individual lane of a flow cell using 40-bp, paired-end reads. Overall, the five libraries yielded a total of 376,243,854 sequencing reads that passed the Illumina GAIIx quality filter.

### Assembly

Each stage-specific library was individually assembled using Velvet (version 1.1.05; [26]) and Oases (version 0.1.22; [27]). For the adult, we used a kmer range of 25–39; for all other stages we used a kmer range of 21–39. For all other assembly parameters, we used the default settings for Velvet and Oases. The individual assemblies were then merged using both Velvet and Oases to produce a single reference transcriptome. The merged assemblies comprise 90,440 contigs.

### Assessment of sequencing coverage

We used a random re-sampling approach to assess how sequencing depth affected recovery of transcripts. All reads from all stages were aligned to the reference file using Bowtie 2 (v. 2.0.0-beta; [68]). The resulting sam file was then parsed with a custom python script (Additional file 10) that randomly selects a given number of reads from the total reads without replacement. This script then returns a file listing the nominal coverage of all contigs, based on the contig length, read length, and number of reads aligned to each contig. The file can then be easily parsed to assess the amount of contigs above each coverage threshold. We evaluated subsets of the total reads ranging from 0 to all of the reads in increments of 50 million. The analysis was performed 3 times for each subset size, except for the 0 and “all” read sets, as the replicates of these sets are guaranteed to be exactly the same each time. For each data point, the standard deviation was calculated, and found to be negligible (all less than 0.1% of the total contigs that pass a given coverage threshold).

### Divergence date estimation

To estimate the divergence between *E. lineata* and *N. vectensis*, we used a molecular clock approach based on the published multi-gene alignment of Erwin et al. [48]. This alignment comprises seven nuclear housekeeping genes (aldolase, methionine adenosyltransferase, ATP synthase beta chain, catalase, elongation factor 1 alpha, triosephosphate isomerase and phosphofructokinase; [37]) and three ribosomal DNAs (5.5S, 18S, and 28S rDNA) from 119 taxa. We restricted our analysis to taxa for which Erwin *et al.*[48] included fossil calibration points (Additional file 2). The resulting alignment included 87 taxa (Additional file 11). We used BLAST searches to identify orthologs of all these genes from *E. lineata*. The *E. lineata* sequences were manually added to the alignment.

The alignment of protein coding and ribosomal genes was input into MrBayes (version 3.1.2 [94], as implemented in the CIPRES Science Gateway, version 3.3), and a phylogeny was estimated using mixed models for the protein and nucleotide partitions of the alignment. We set up one run of four chains using two unlinked GTR + gamma models: an amino acid GTR + gamma model was applied to the amino acid partition, and a nucleotide GTR + gamma model was applied to the rDNA partition. The shape of the gamma distribution was estimated using four rate categories for each partition. Chains were allowed to run for 1,000,000 generations, with a burn-in of 25%, and sampling every 5,000. The resulting tree for the full set of 87 taxa can be viewed in Additional file 2.

Bayesian estimation of divergence dating was carried out using the program Phylobayes (version 3.3b; [95,96]). The current iteration of Phylobayes does not support mixed (protein and nucleotide) datasets for divergence dating, so we followed the example established by Erwin et al. [48] and used just the protein-coding characters for the divergence dating analysis. The chronogram resulting from Phylobayes is available in Additional file 2.

### Transcriptome annotation

All 90,440 contigs were compared against the non-redundant (NR) database on NCBI using BLASTx at a threshold Expect value of 1E-03. Contigs with no match were BLASTed against a database of noncoding nucleotides on the NONCODE database [90] to search for homology to transcribed RNAs that are not translated into protein.

From the BLAST results, the taxonomic source of the top five hits obtained for each contig were stored in EdwardsiellaBase. To estimate the phylogenetic origin of sequences in the *E. lineata* transcriptome, protein lists were downloaded from NCBI using a series of scripts (Additional file 12) for a selection of taxonomic categories encompassing taxa of increasingly distant evolutionary relationship to *E. lineata*. The taxonomic categories used were: (1) *N. vectensis*, (2) Cnidaria excluding *N. vectensis*, (3) Bilateria, (4) Metazoa excluding Cnidaria and Bilateria, (5) Eukaryota excluding Metazoa, (6) Archaea, (7) Eubacteria, and (8) viruses. For this search, we also used BLASTx at a threshold Expect value of 1E-03.

GO terms were assigned to contigs through the Blast2GO servers after importing the BLAST results. Production of informative graphs about the GO data was generated through analysis of the data via a custom Python script (Additional file 5) which parses a file (gene_ontology.obo) from the Gene Ontology ftp site containing information about each node and its parent(s) and children. From this, information about the GO hierarchy is parsed by the script, and stored temporarily. Using the recovered GO data, and a starting node in the hierarchy, the script then looks for nodes below the starting node in the hierarchy for which GO data was recovered in the transcriptome data in order to determine the coverage of the sub-hierarchy. With this script, a user can identify all the contigs associated with a particular GO term and its subtree. In our analysis, we grouped all contigs according to the highest sub-category under the principal GO categories: Biological Process, Cellular Component, and Molecular Function (Figure 8; Additional file 6).

Blast2GO annotated contigs with Enzyme Commission (E.C.) numbers when applicable. Available E.C. numbers for *N. vectensis* were obtained through the Kyoto Encyclopedia of Genes and Genomes (KEGG; [50]). The E.C. numbers for *E. lineata* and *N. vectensis* were compared to see which enzymes were in both sets, and which were exclusive to one anemone or the other. Enzymes were then formatted, and cross-referenced to an edge list file from the interactive tree of life to produce a file (Additional file 13), which was uploaded to the iPath2.0 program for visualization (Figure 9; Additional file 7; [51]).

### Recovery of gene families from *E. lineata*

We compiled FASTA files containing published protein sequences from *N. vectensis* for bHLH-PAS genes, deiodinases, Fox genes, LIM homeodomains, minicollagens, nuclear receptors, Sox genes, and Wnts. We then queried the *E. lineata* transcriptome with these sequences using tBLASTn. The top 10 hits from *E. lineata* were retained from each query. These were used to perform reciprocal BLASTx searches versus the FASTA file containing the protein sequences from *N. vectensis* to verify that each *E. lineata* sequence is most similar to the original query sequence. This sequence of BLAST searches was performed using a custom Python script (Additional file 14). In the case of all gene families except minicollagens (which are unique to Cnidaria), predicted protein sequences were obtained from *N. vectensis*, *E. lineata*, and *Homo sapiens*. Sequences were aligned using MUSCLE [97], and amino acid characters with gaps were removed from the alignment. The resulting gap-free alignments were then analyzed using ProtTest (v.3; [98]) to determine the best-fit model of amino acid replacement according to the Akaike Information Criterion. Maximum-likelihood phylogenies were estimated from the edited alignments using the default parameters of RaxML-HPC2 [99] as implemented at the CIPRES Science Gateway [100]. To evaluate the support for interior nodes, 1000 replicates of the bootstrap were performed [101].

A complete 18S rDNA transcript was recovered from the specimens sequenced for this study via a BLAST search of EdwardsiellaBase using *N. vectensis* 18S rDNA as a query sequence. This 18S sequence was then aligned to published 18S sequences for eight other edwardsiid anemones using the default parameters of MUSCLE [97]. Gaps and poorly-aligned regions were removed with Gblocks [102]. The edited alignment is available in Additional file 1. A maximum-likelihood phylogeny was estimated from this edited alignment using the default parameters of RaxML-HPC2 [99] as implemented at the CIPRES Science Gateway [100]. To evaluate the support for interior nodes, 1000 replicates of the bootstrap were performed [101].

For all protein families examined here, we used MEME (Multiple Expectation Maximization for Motif Elicitation; [103]) to identify conserved motifs in orthologs and paralogs from the various species sampled. Motif searches were performed under the following settings: maximum number of motifs = 10; occurrences of a single motif = any number; minimum length of a motif = 5 amino acids; maximum length of a motif = 100. Conserved motifs are depicted in the relevant figures to the right of each gene’s name (Figures 10, 12; Additional file 8).

### Database construction

EdwardsiellaBase is a relational database constructed in PostgreSQL (version 8.4.4). It houses the *E. lineata* contigs generated in this study in addition to the results from a number of bioinformatics analyses performed on these contigs. The database structure and entity relationships are depicted in Additional file 9. Files to construct the database were prepared and parsed from resulting data, and available data from NCBI, Expasy, and amiGO. Web pages are generated in real time using Python scripts that query the database through the pgdb module for Python. The BLAST suite of programs (v. 2.2.24+) is installed on the server, and is run with a query against specific BLAST-formatted databases using the subprocess module of Python. The raw sequencing reads were aligned to the assembled contigs and preloaded into a file structure that allows the user to quickly locate and display alignment to a contig of interest through JBrowse (v. 1.7.6; [69,70]).

## Competing interests

The authors declare that they have no competing interests.

## Authors’ contributions

DJS performed field collection of specimens and developmental manipulations, photography, RNA isolation, cDNA library synthesis. DJS and TJL performed divergence dating analysis. TJL and BRG assembled the reference transcriptome. BRG performed the saturation curve and metabolic pathway analysis. TJL and BRG carried out BLAST and GO analyses. TJL, ALB, and DJS performed gene family recovery. AMR and DJS carried out phylogenetic analyses. DJS, LD, AL, and JRF carried out MEME analyses. TJL and BRG constructed the database and the internet interface for Edwardsiellabase. DJS, TJL, BRG and AMR and JRF contributed to production of the figures and drafting of the manuscript. JRF oversaw the study design, data analysis, production of figures, and the writing. All authors read and approved the final manuscript.

## Supplementary Material

Additional file 1 **Edwardsiidae_18S_alignment. **A nexus file containing 18S rDNA sequences from six species of edwardsiid anemones and one outgroup taxon, *Metridium senile*. Click here for file

Additional file 2 **DivergenceDating__trees-clock-calib_85taxa.** A pdf file containing a table of fossil dates used to calibrate the molecular clock, the phylogenetic tree of 85 taxa from MrBayes, and the chronogram from Phylobayes. Click here for file

Additional file 3 **ReadsMappingToContigsProducingBlastHits.** Bar graphs depicting (A) the average number of sequencing reads and (B) the overall number of sequencing reads that map to contigs that produce BLAST hits versus those contigs that do not produce BLAST hits. Click here for file

Additional file 4 **LengthOfContigsProducingBlastHits.** A histogram depicting the frequency of a range of contig lengths for contigs that produce BLAST hits versus contigs that do not produce BLAST hits. Click here for file

Additional file 5 **GeneOntologyExtractionScript.** A custom python script that extracts gene ontology terms from a gene_ontology.obo file. Click here for file

Additional file 6 **GeneOntology.** Two bar graphs depicting the recovery of possible GO terms under each of the primary subcategories of “Biological Process” and “Cellular Component”. The bars depict the total number of terms in each subcategory (grey), the number of subcategories recovered in *Edwardsiella* (dark blue), and the number of subcategories recovered in *Nematostella* (light blue) using a Log scale. The absolute numbers are provided on or above each bar. Click here for file

Additional file 7 **CompleteMetabolicNetwork.** The complete collection of metabolic pathways as represented by iPath. The nodes represent metabolites, and the edges represent metabolic transformations. Green edges indicate pathways that were found in both *N. vectensis* and *E. lineata*. Red pathways were only found in *N. vectensis*, and yellow pathways were only found in *E. lineata*. Gray and black edges indicate pathways that were not found in either anemone, in the case of gray edges because no Enzyme Commission numbers map to these edges, and thus they were impossible to detect in our analysis. Click here for file

Additional file 8 **MaximumLikelihoodGeneTrees.** Maximum likelihood gene trees for bHLH-PAS, deiodinases, LIM homeodomains, minicollagens, and nuclear receptors. With the exception of minicollagens, each gene family analysis was based on protein sequences from deuterostome (human), protostome (*Platynereis dumerilii*), and cnidarian (*Nematostella vectensis; Edwardsiella lineata*) lineages. The location of conserved motifs for each protein sequence is also shown. Details of individual phylogenetic analyses are contained within the file. Click here for file

Additional file 9 **EdwardsiellaBaseEntityRelationship.** A graphic depicting the database structure and entity relationships of EdwardsiellaBase. Details are contained within the file. Click here for file

Additional file 10 **SequencingSaturationCurveScript.** A custom python script that randomly selects a given number of reads from the total reads without replacement and determines the fraction of the overall assembly that passes a certain coverage threshold. Click here for file

Additional file 11 **DivergenceDating_Alignment_85taxa.** A Phylip file containing an alignment of amino acid sequences from seven concatenated proteins from 85 different taxa. Click here for file

Additional file 12 **NCBITaxonRestrictedRetrieval.** A collection of custom python scripts that perform iterative, taxonomically restricted BLAST searches against the sequences housed at NCBI in an attempt to infer the phylogenetic generality and evolutionary origin of gene sequences. Click here for file

Additional file 13 **iPathInput.** An edge list file from the interactive tree of life that can be read by iPath2.0 to visualize the presence or absence of metabolic pathways in *E. lineata* and *N. vectensis*. Click here for file

Additional file 14 **GeneFamilyRecoveryScript.** A script to detect gene families in transcriptome assemblies using biopython and BLAST. Inputs include a fasta file of protein sequences and fasta files for multiple different transcriptome assemblies. The script identifies the presence or absence of each of the proteins in each of the assemblies, thus providing an easy way to compare the gene repertoire of multiple different assemblies. Click here for file
