# Supplementary material for: Production of a reference transcriptome and transcriptomic database (EdwardsiellaBase) for the lined sea anemone, Edwardsiella lineata, a parasitic cnidarian
Source: BMC Genomics. 2014 Jan 28;15:71. doi: 10.1186/1471-2164-15-71 (PMC3909931; doi:10.1186/1471-2164-15-71)
Supplement: Additional file 2 — DivergenceDating__trees-clock-calib_85taxa. A pdf file containing a table of fossil dates used to calibrate the molecular clock, the phylogenetic tree of 85 taxa from MrBayes, and the chronogram from Phylobayes. [file 1471-2164-15-71-S2.pdf]

Taxon 1	Taxon 2	Upper Limit	Lower Limit	Source
Dendraster	Encope	-1	50	Peterson et al., 2004
Dendraster	Eucidaris	-1	255	Peterson et al., 2004
Dendraster	Asterina	-1	480	Peterson et al., 2004
Dendraster	Antedon	525	485	Peterson et al., 2004
Dendraster	Saccoglossus	565	-1	Peterson et al., 2008; Lieu et al., 2010
M_edulis	M_califorianus	-1	20	Peterson et al., 2004
M_edulis	Modiolus	-1	235	Peterson et al., 2004
M_edulis	Nucula	-1	485	Peterson et al., 2004
Haliotis	Crepidula	-1	500	Peterson and Butterfield, 2005
M_edulis	Crepidula	548	530	Peterson et al., 2008
Lestes	Enallagma	-1	120	Peterson et al., 2004
Drosophila	Aedes	295	235	Peterson et al., 2004; Benton and Donoghue, 2007
Anopheles	Enallagma	-1	325	Peterson et al., 2004
Drosophila	Priapulus	-1	522	Benton and Donoghue, 2007
Drosophila	Daphnia	-1	500	Walossek, 1995
Daphnia	Rhipicephalus	-1	515	Rota-Stabelli et al., 2010; Maloof et al., 2010
Anolis	Gallus	299	259	Benton and Donoghue, 2007
Homo	Gallus	330	312	Benton and Donoghue, 2007
Homo	Xenopus	350	330	Benton and Donoghue, 2007
Homo	Monodelphis	138	124	Benton and Donoghue, 2007
Homo	Rattus	100	61	Benton and Donoghue, 2007
Homo	Danio	421	416	Benton and Donoghue, 2007
Dani	Tetraodon	165	149	Benton and Donoghue, 2007
Geodia	Verongula	713	-1	Peterson et al., 2007' Sperling et al., 2010
Nematostella	Acropora	-1	520	Hou et al., 2005; Zhang et al, 2008
Nematostella	Hydra	-1	503	Cartwright et al., 2007

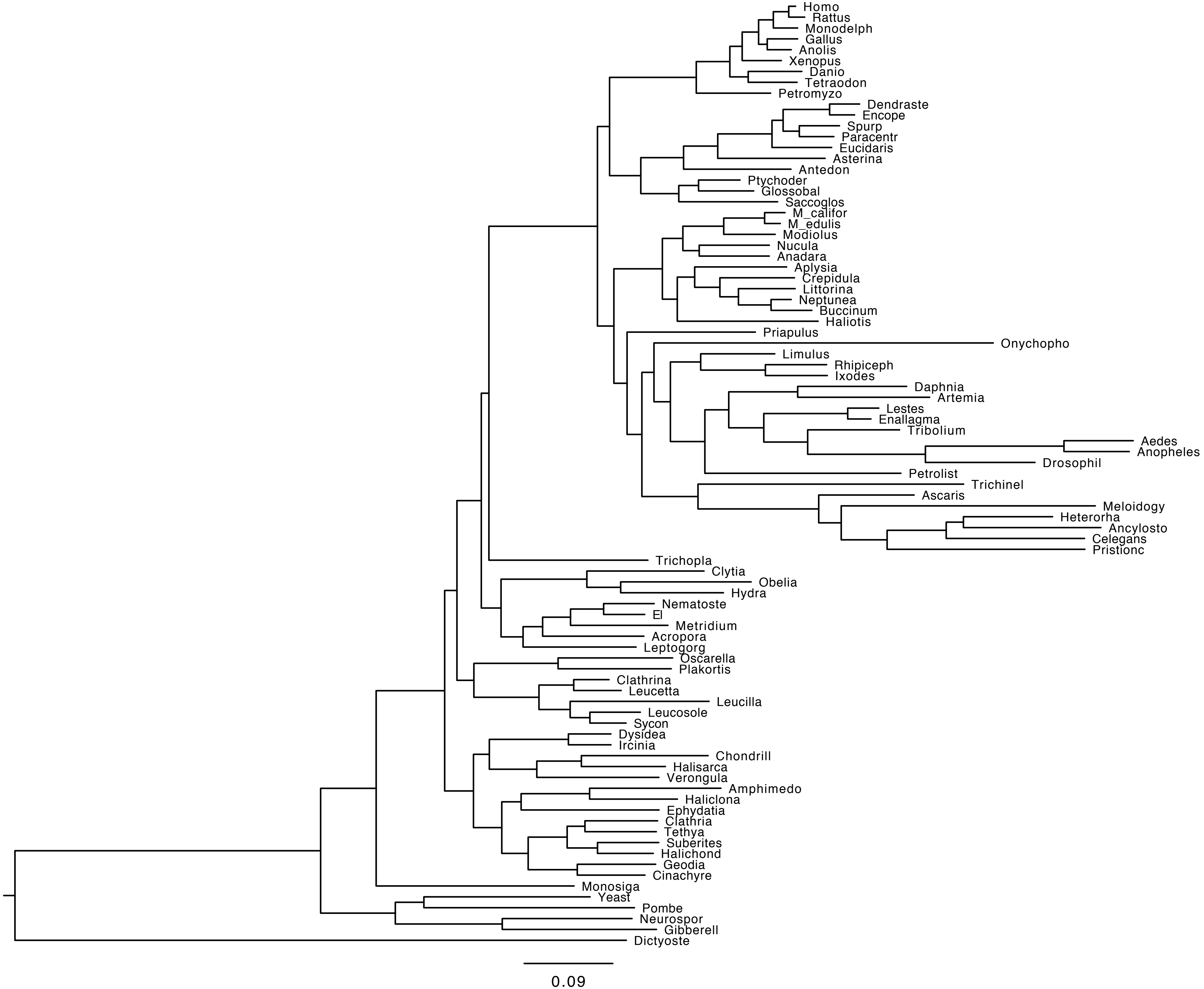
Homo
Rattus
Monodelph
Gallus
Anolis
Xenopus
Danio
Tetraodon
Petromyzo
Dendraste
Encope
Spurp
Paracentr
Eucidaris
Asterina
Antedon
Ptychoder
Glossobal
Saccoglos
M_califor
M_edulis
Modiolus
Nucula
Anadara
Aplysia
Crepidula
Littorina
Neptunea
Buccinum
Haliotis
Priapulus
Onychopho
Limulus
Rhipiceph
Ixodes
Daphnia
Artemia
Lestes
Enallagma
Tribolium
Aedes
Anopheles
Drosophil
Petrolist
Trichinel
Ascaris
Meloidogy
Heterorha
Ancylosto
Celegans
Pristionc
Trichopla
Clytia
Obelia
Hydra
Nematoste
El
Metridium
Acropora
Leptogorg
Oscarella
Plakortis
Clathrina
Leucetta
Leucilla
Leucosole
Sycon
Dysidea
Ircinia
Chondrill
Halisarca
Verongula
Amphimedo
Haliclona
Ephydatia
Clathria
Tethya
Suberites
Halichond
Geodia
Cinachyre
Monosiga
Yeast
Pombe
Neurospor
Gibberell
Dictyoste
0.09

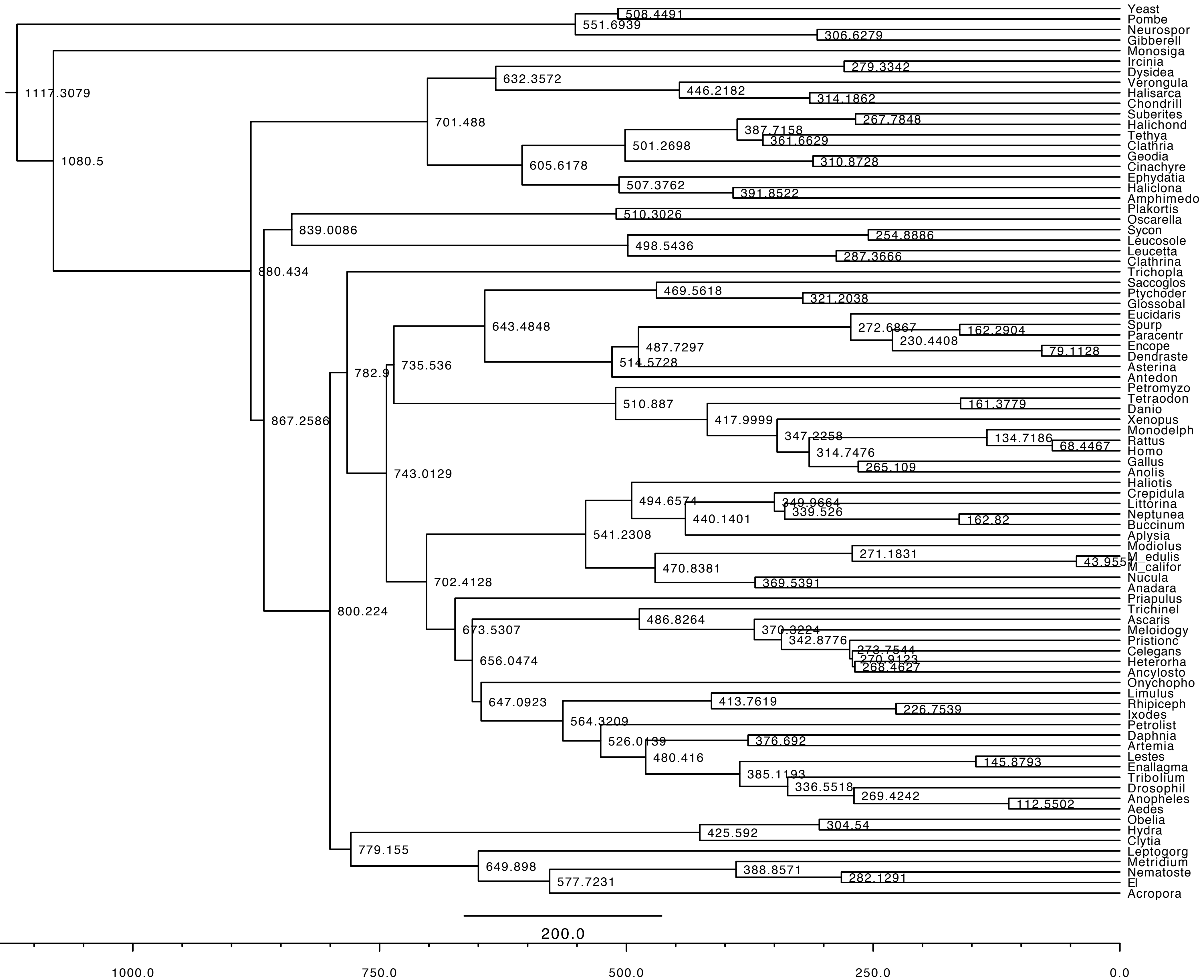
Yeast
Pombe
Neurospor
Gibberell
Monosiga
Ircinia
Dysidea
Verongula
Halisarca
Chondrill
Suberites
Halichond
Tethya
Clathria
Geodia
Cinachyre
Ephydatia
Haliclona
Amphimedo
Plakortis
Oscarella
Sycon
Leucosole
Leucetta
Clathrina
Trichopla
Saccoglos
Ptychoder
Glossobal
Eucidaris
Spurp
Paracentr
Encope
Dendraste
Asterina
Antedon
Petromyzo
Tetraodon
Danio
Xenopus
Monodelph
Rattus
Homo
Gallus
Anolis
Haliotis
Crepidula
Littorina
Neptunea
Buccinum
Aplysia
Modiolus
M_edulis
M_califor
Nucula
Anadara
Priapulus
Trichinel
Ascaris
Meloidogy
Pristionc
Celegans
Heterorha
Ancylosto
Onychopho
Limulus
Rhipiceph
Ixodes
Petrolist
Daphnia
Artemia
Lestes
Enallagma
Tribolium
Drosophil
Anopheles
Aedes
Obelia
Hydra
Clytia
Leptogorg
Metridium
Nematoste
El
Acropora
1117.3079
1080.5
551.6939
508.4491
306.6279
880.434
701.488
632.3572
279.3342
446.2182
314.1862
605.6178
501.2698
387.7158
267.7848
361.6629
310.8728
507.3762
391.8522
839.0086
510.3026
498.5436
254.8886
287.3666
867.2586
782.9
735.536
643.4848
469.5618
321.2038
514.5728
487.7297
272.6867
162.2904
230.4408
79.1128
510.887
161.3779
417.9999
347.2258
314.7476
134.7186
68.4467
265.109
800.224
743.0129
702.4128
541.2308
494.6574
349.9664
440.1401
339.526
162.82
470.8381
271.1831
43.955
369.5391
673.5307
656.0474
486.8264
370.3224
342.8776
273.7544
270.9123
268.4627
647.0923
413.7619
226.7539
564.3209
526.0139
376.692
480.416
145.8793
385.1193
336.5518
269.4242
112.5502
779.155
425.592
304.54
649.898
388.8571
577.7231
282.1291
200.0
1000.0
750.0
500.0
250.0
0.0
