# Supplementary material for: Production of a reference transcriptome and transcriptomic database (EdwardsiellaBase) for the lined sea anemone, Edwardsiella lineata, a parasitic cnidarian
Source: BMC Genomics. 2014 Jan 28;15:71. doi: 10.1186/1471-2164-15-71 (PMC3909931; doi:10.1186/1471-2164-15-71)
Supplement: Additional file 3 — ReadsMappingToContigsProducingBlastHits. Bar graphs depicting (A) the average number of sequencing reads and (B) the overall number of sequencing reads that map to contigs that produce BLAST hits versus those contigs that do not produce BLAST hits. [file 1471-2164-15-71-S3.pdf]

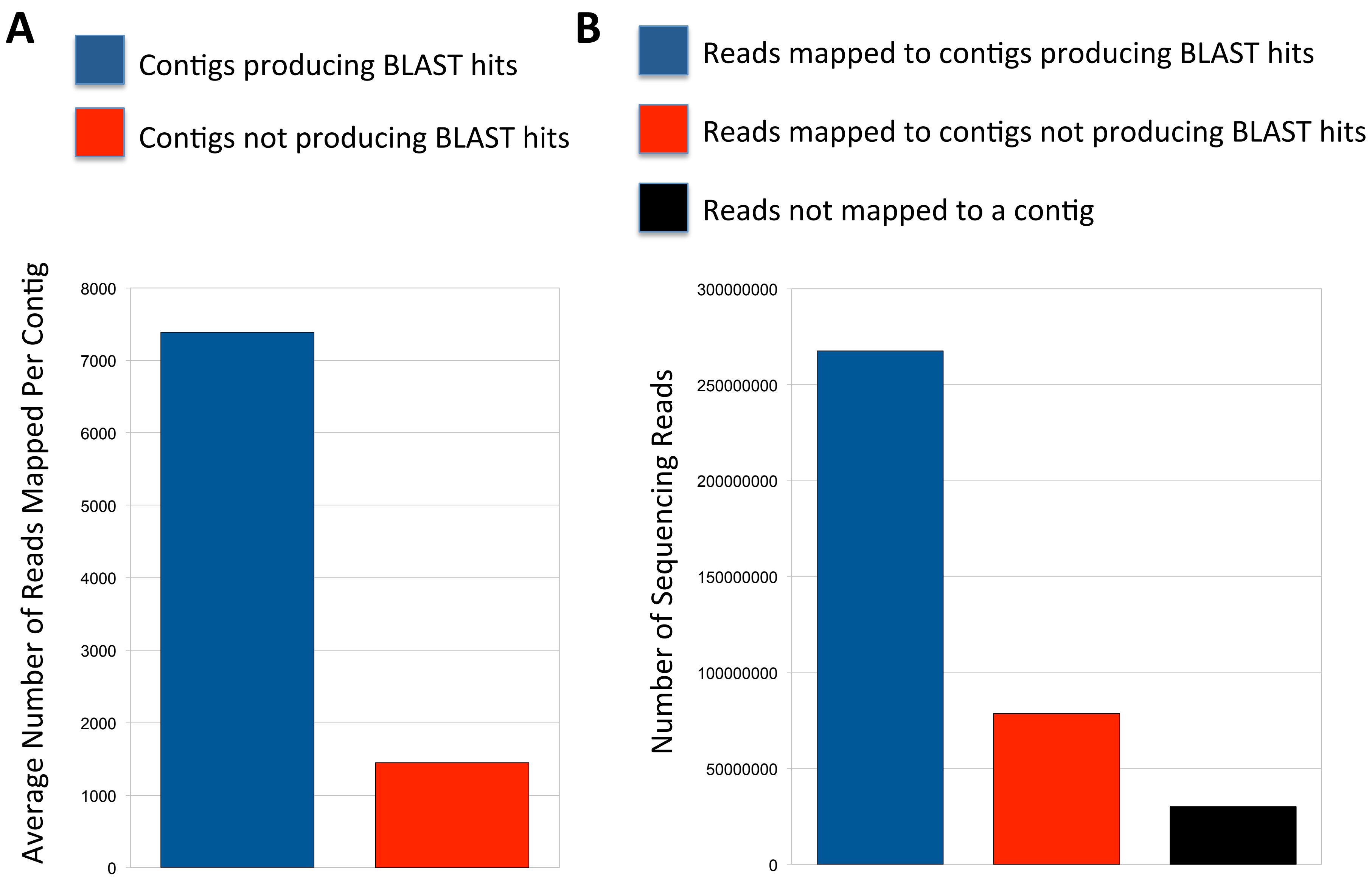
A
Contigs producing BLAST hits
Contigs not producing BLAST hits
Average Number of Reads Mapped Per Contig
8000
7000
6000
5000
4000
3000
2000
1000
0
B
Reads mapped to contigs producing BLAST hits
Reads mapped to contigs not producing BLAST hits
Reads not mapped to a contig
Number of Sequencing Reads
300000000
250000000
200000000
150000000
100000000
50000000
0
