# Supplementary figures and images for: Production of a reference transcriptome and transcriptomic database (EdwardsiellaBase) for the lined sea anemone, Edwardsiella lineata, a parasitic cnidarian
Source: BMC Genomics. 2014 Jan 28;15:71. doi: 10.1186/1471-2164-15-71 (PMC3909931; doi:10.1186/1471-2164-15-71)

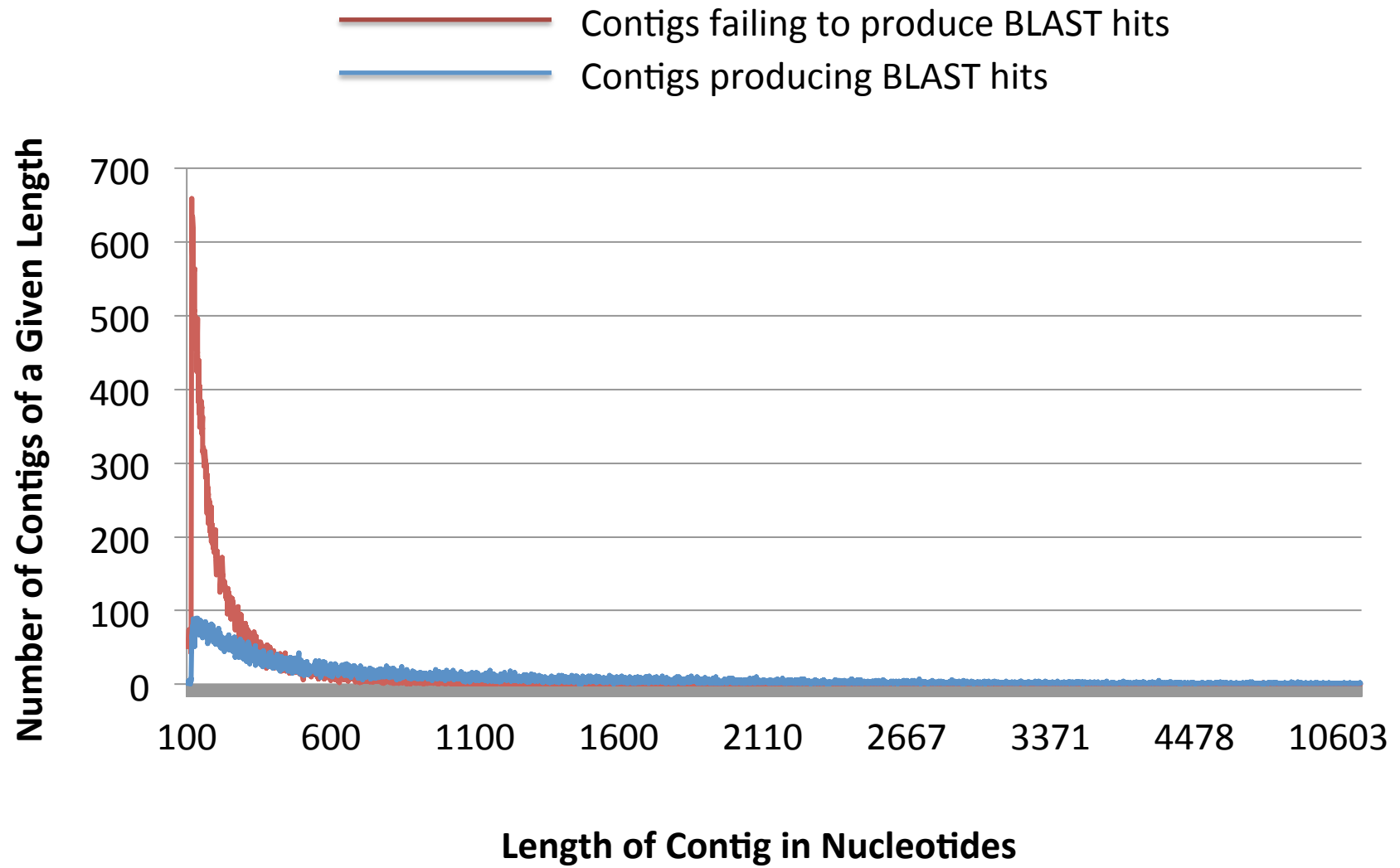

Supplement: Additional file 4 — LengthOfContigsProducingBlastHits. A histogram depicting the frequency of a range of contig lengths for contigs that produce BLAST hits versus contigs that do not produce BLAST hits. [file 1471-2164-15-71-S4.pdf]

# Biological Process

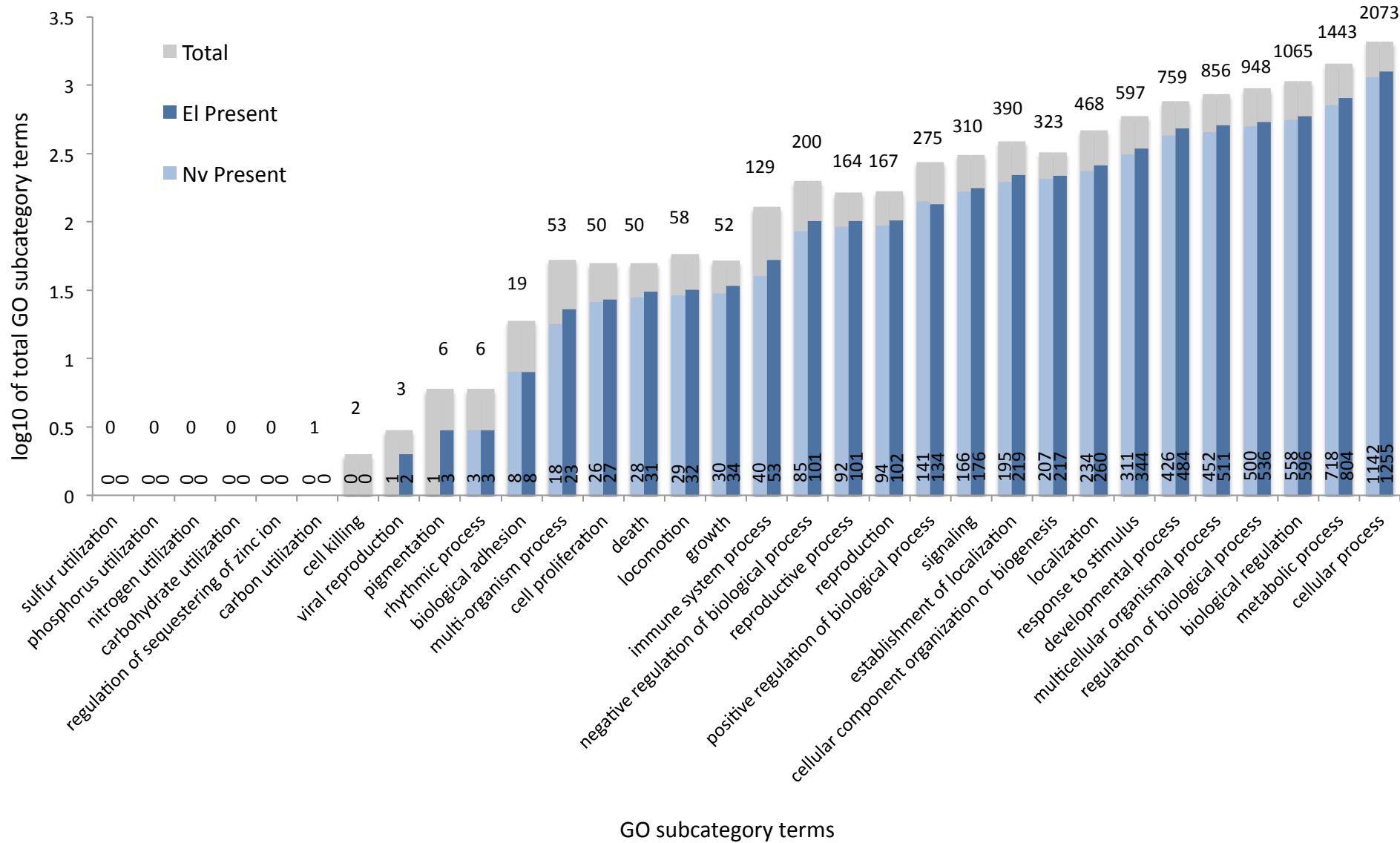

# Cellular Component

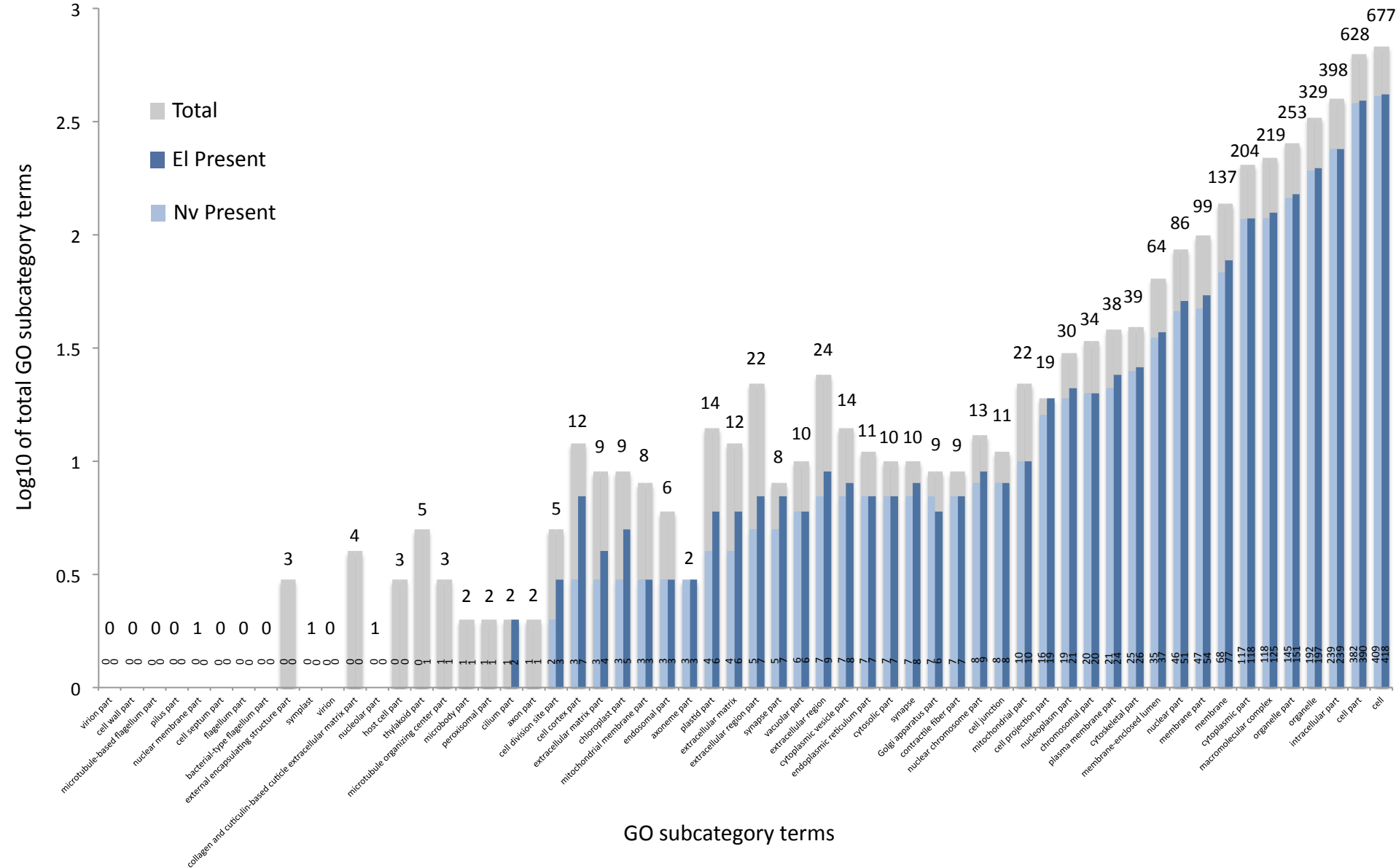

Supplement: Additional file 6 — GeneOntology. Two bar graphs depicting the recovery of possible GO terms under each of the primary subcategories of “Biological Process” and “Cellular Component”. The bars depict the total number of terms in each subcategory (grey), the number of subcategories recovered in Edwardsiella (dark blue), and the number of subcategories recovered in Nematostella (light blue) using a Log scale. The absolute numbers are provided on or above each bar. [file 1471-2164-15-71-S6.pdf]

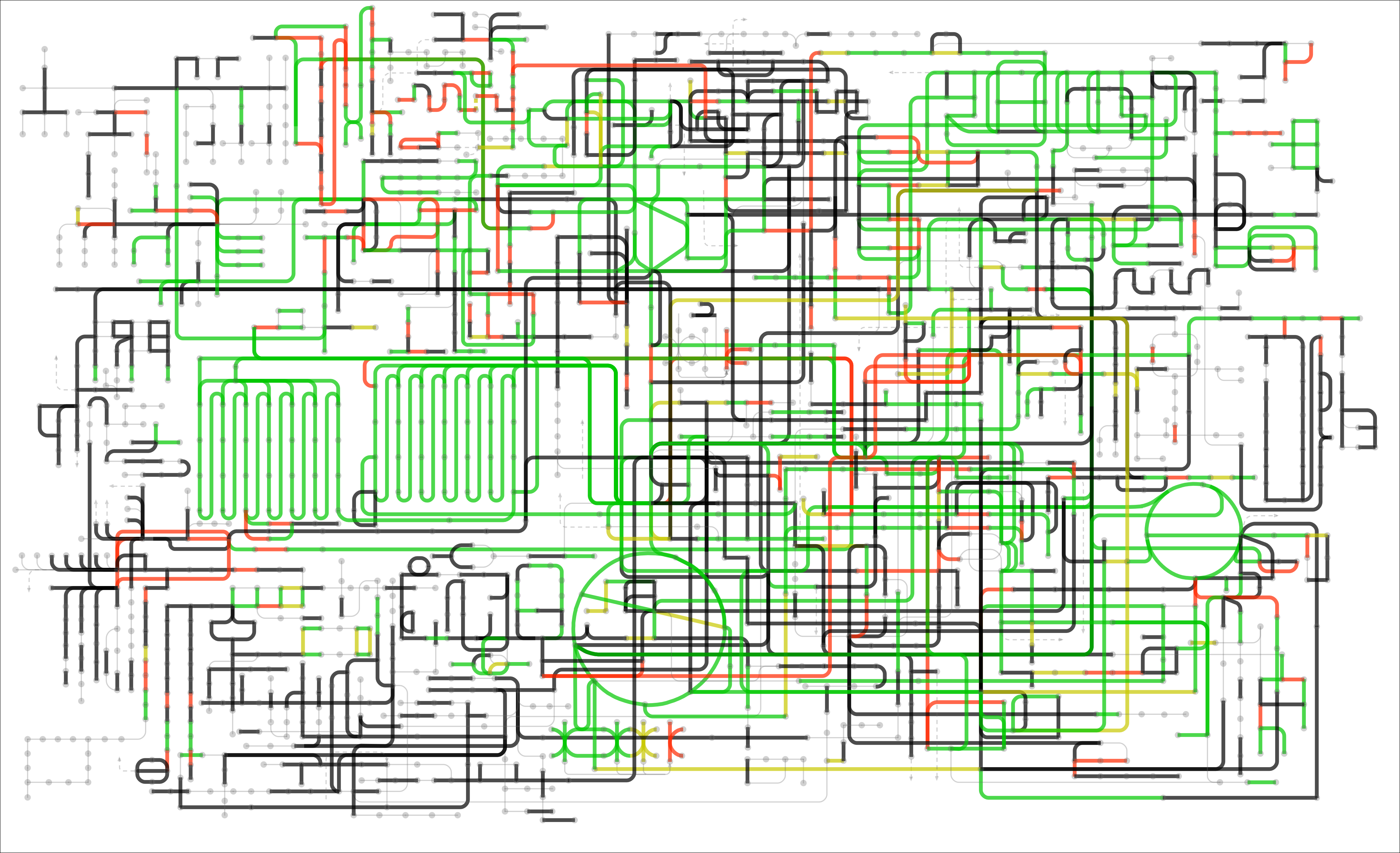

Supplement: Additional file 7 — CompleteMetabolicNetwork. The complete collection of metabolic pathways as represented by iPath. The nodes represent metabolites, and the edges represent metabolic transformations. Green edges indicate pathways that were found in both N. vectensis and E. lineata. Red pathways were only found in N. vectensis, and yellow pathways were only found in E. lineata. Gray and black edges indicate pathways that were not found in either anemone, in the case of gray edges because no Enzyme Commission numbers map to these edges, and thus they were impossible to detect in our analysis. [file 1471-2164-15-71-S7.png]

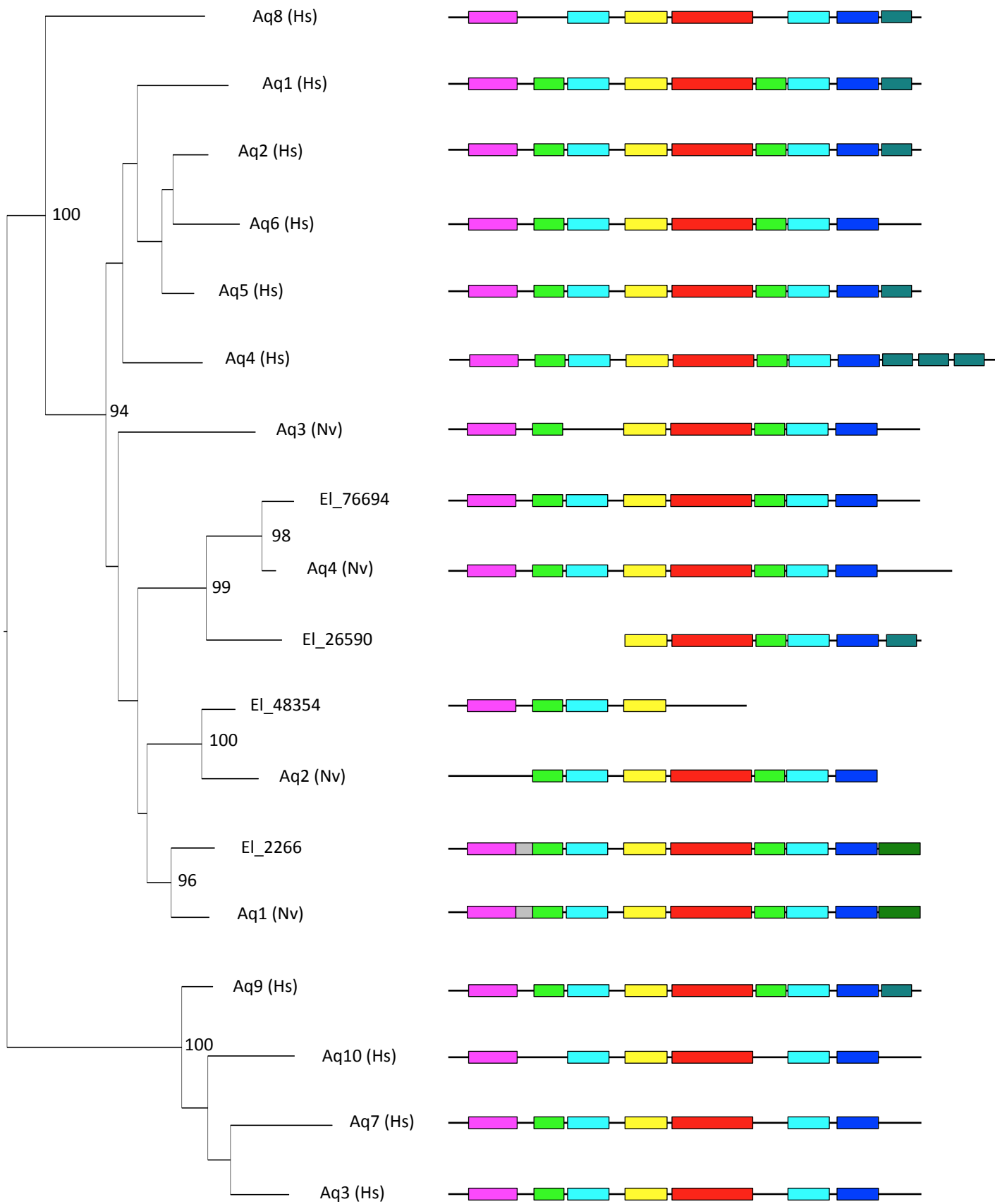

0.2

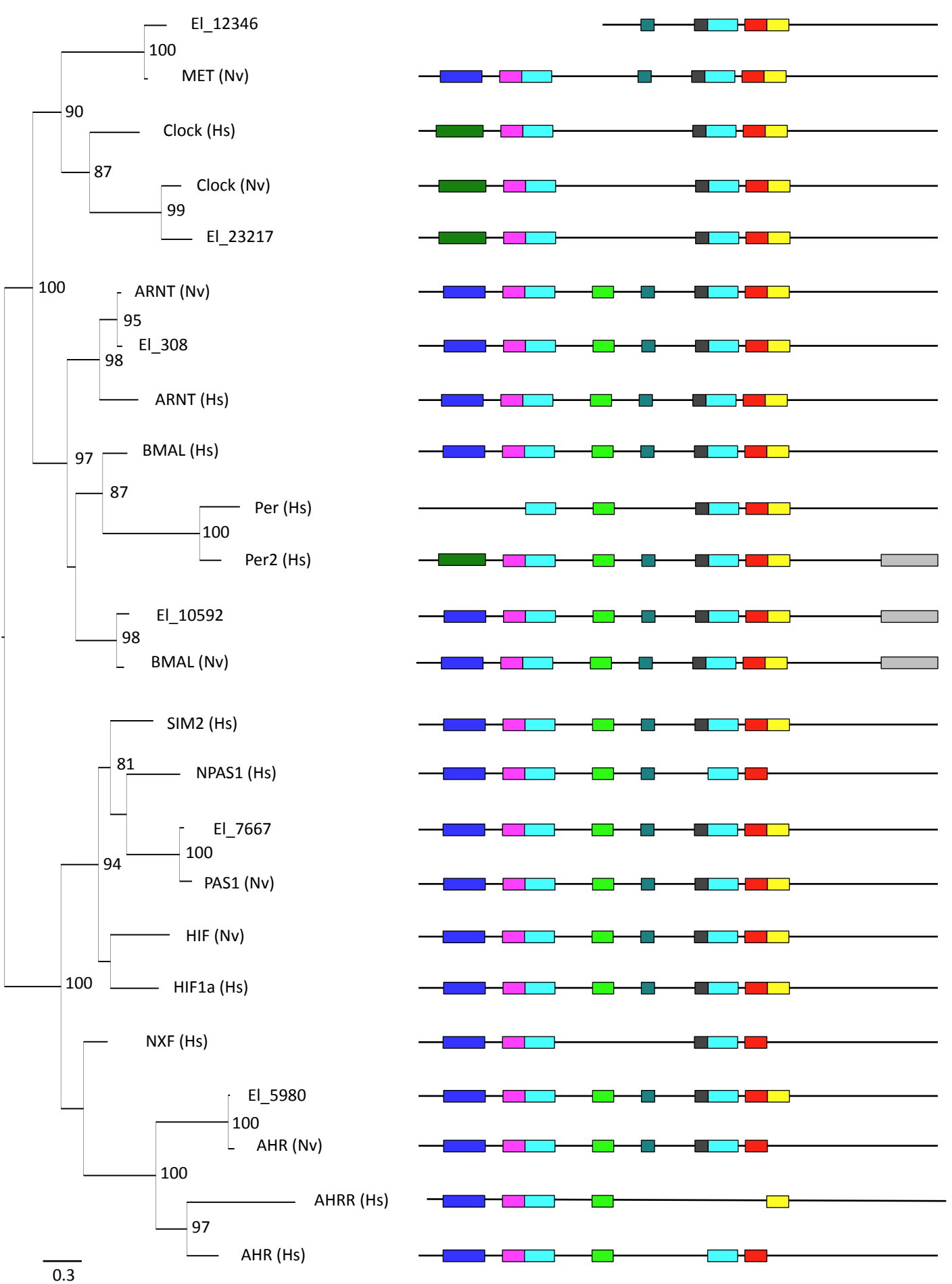

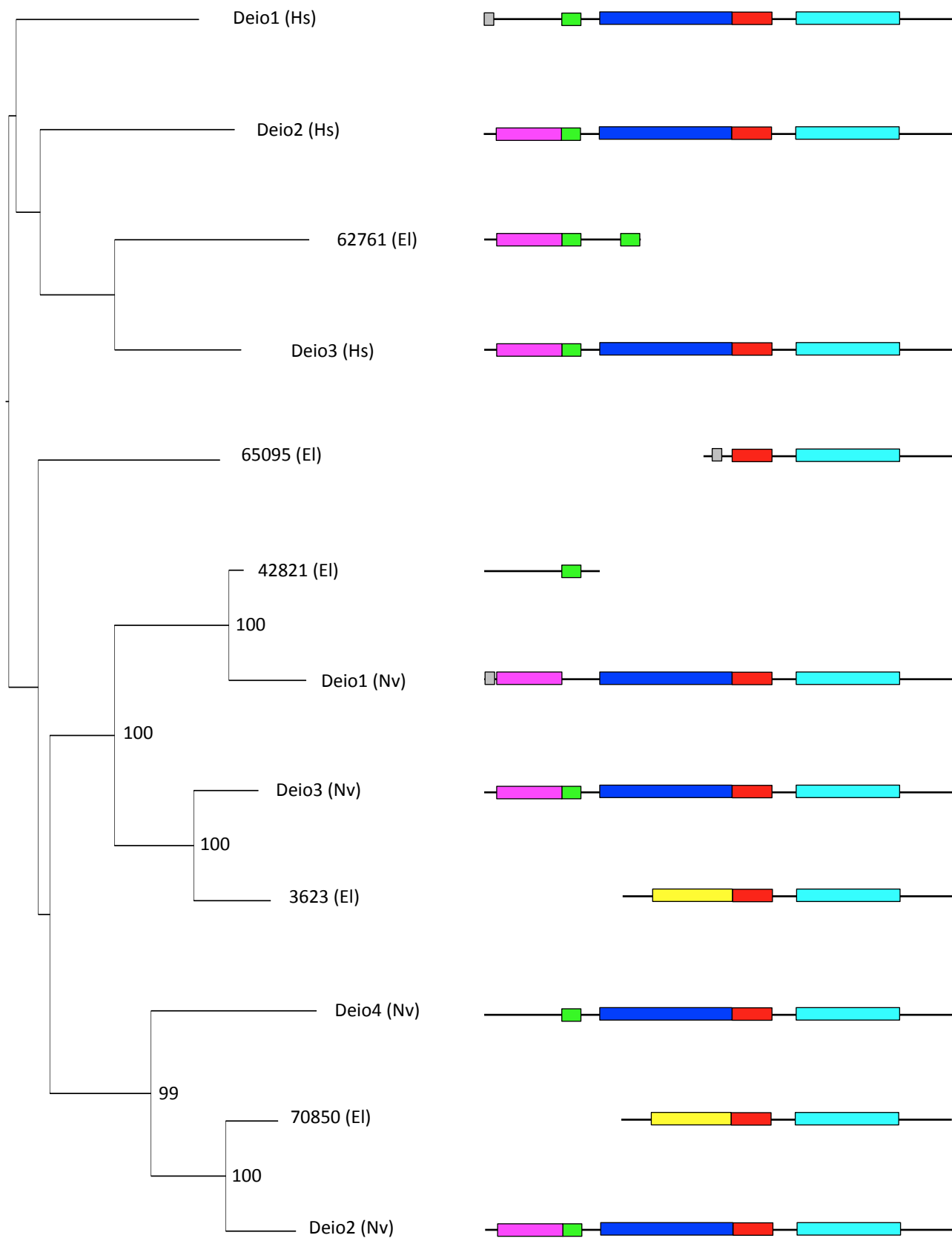

0.1

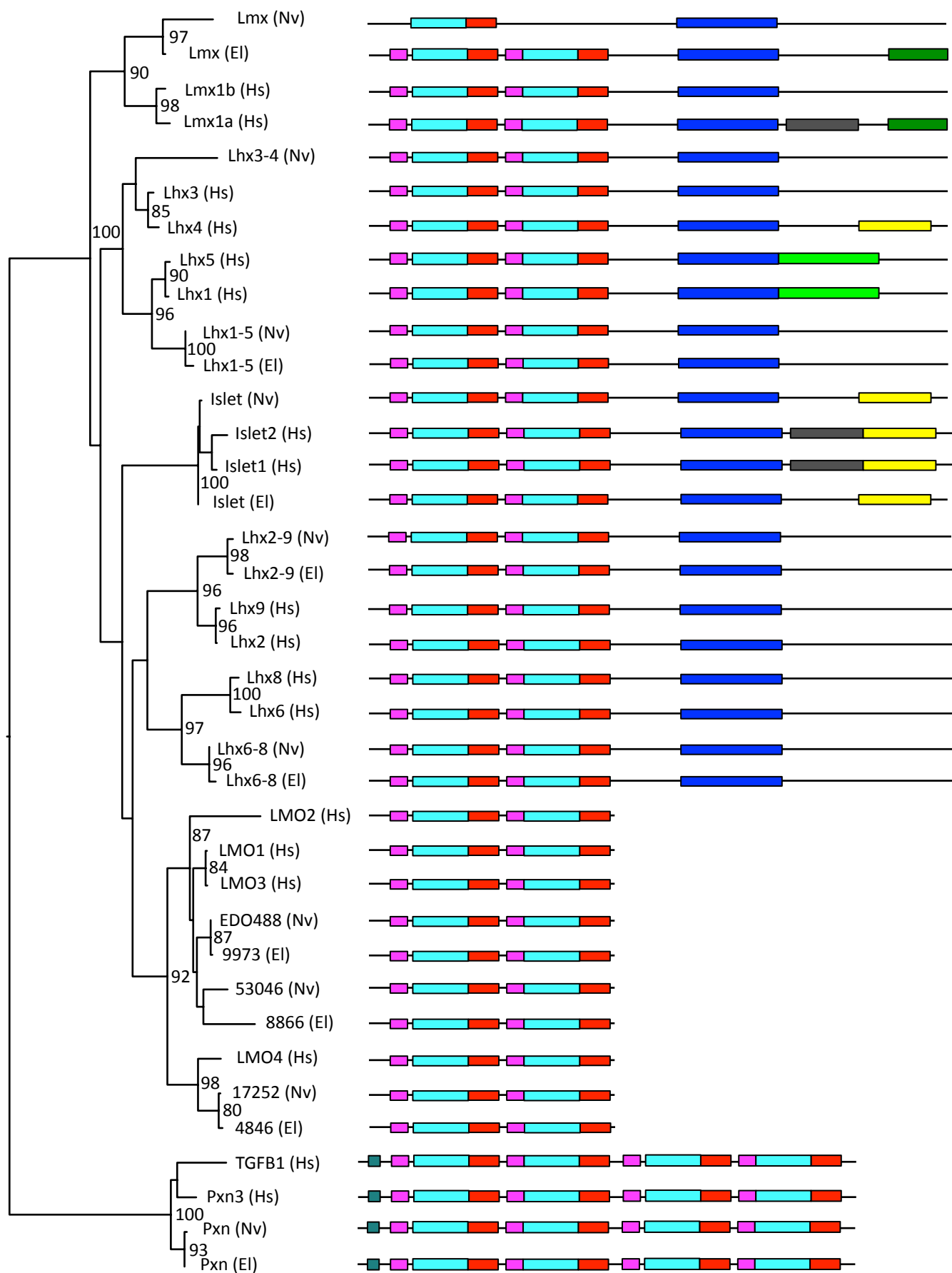

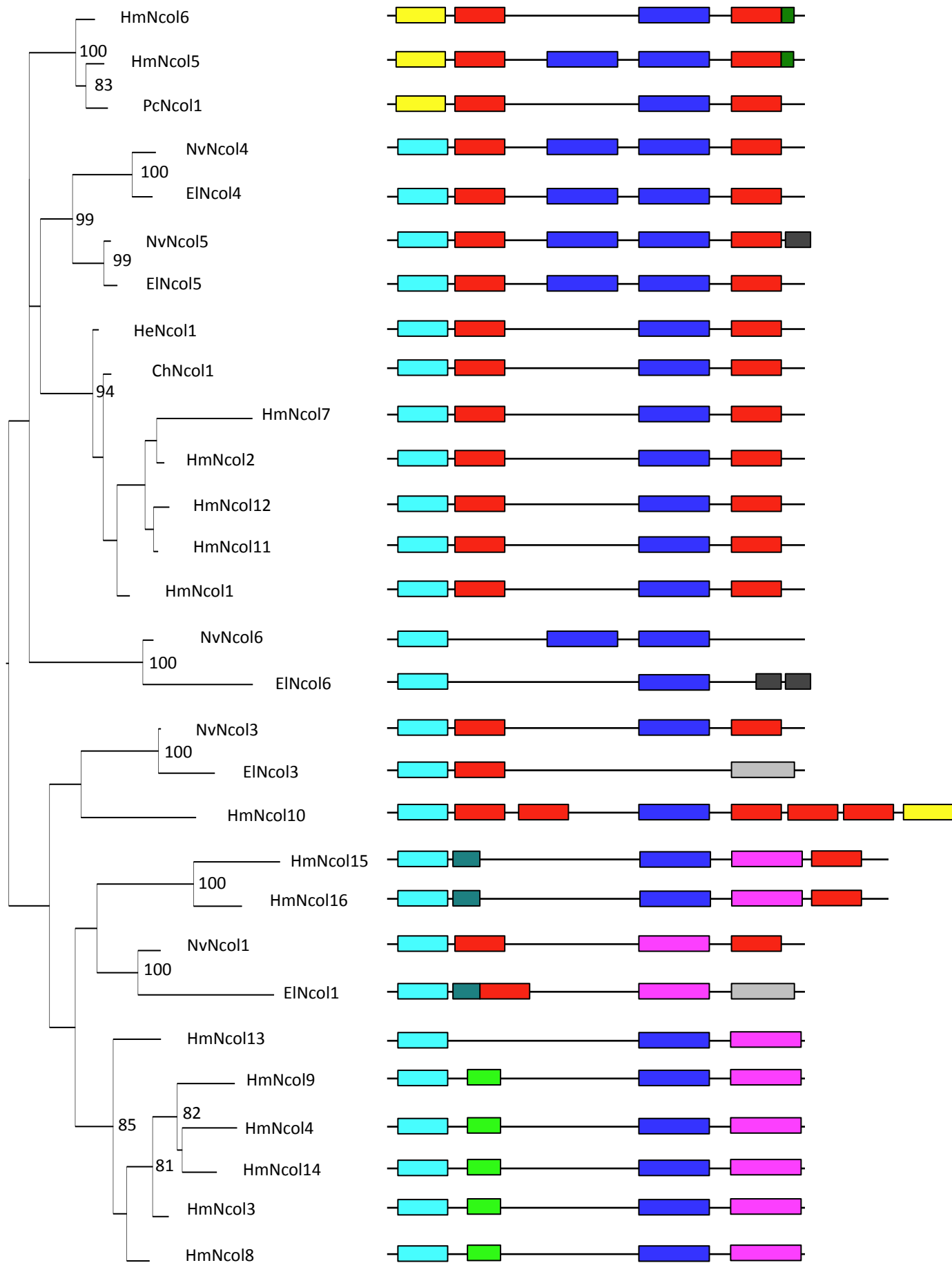

0.3

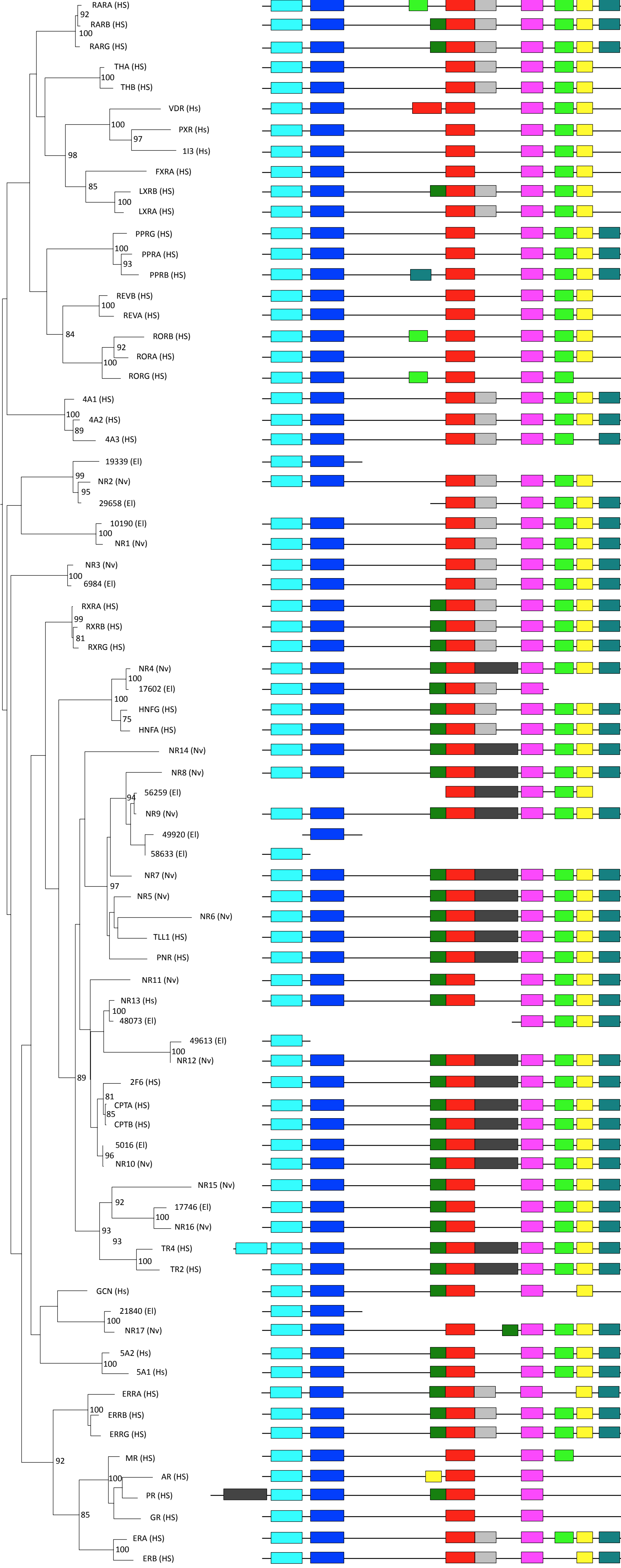

Supplement: Additional file 8 — MaximumLikelihoodGeneTrees. Maximum likelihood gene trees for bHLH-PAS, deiodinases, LIM homeodomains, minicollagens, and nuclear receptors. With the exception of minicollagens, each gene family analysis was based on protein sequences from deuterostome (human), protostome (Platynereis dumerilii), and cnidarian (Nematostella vectensis; Edwardsiella lineata) lineages. The location of conserved motifs for each protein sequence is also shown. Details of individual phylogenetic analyses are contained within the file. [file 1471-2164-15-71-S8.pdf]

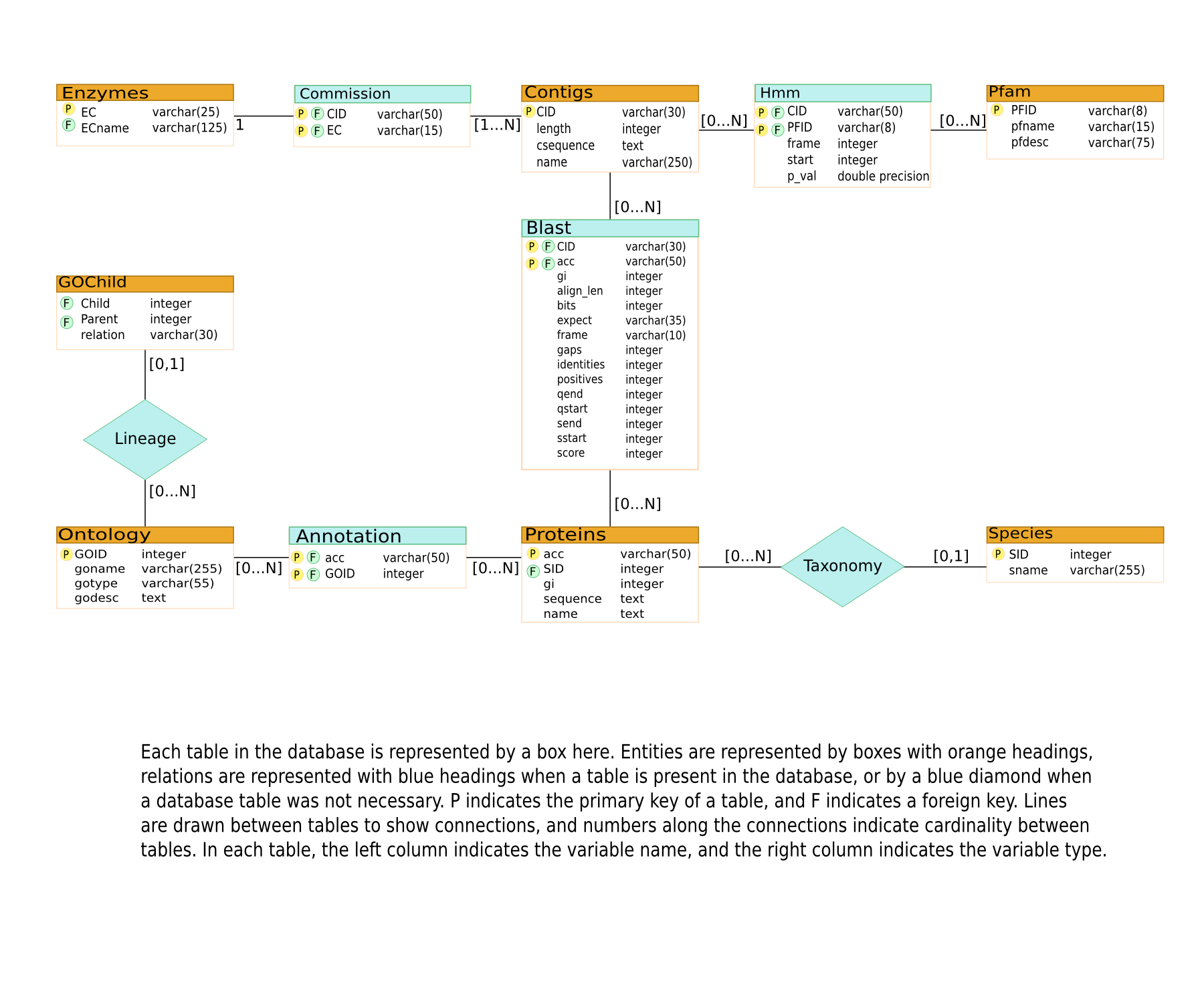

Supplement: Additional file 9 — EdwardsiellaBaseEntityRelationship. A graphic depicting the database structure and entity relationships of EdwardsiellaBase. Details are contained within the file. [file 1471-2164-15-71-S9.png]
